# The Epidemiological and Mechanistic Understanding of the Neurological Manifestations of COVID-19: A Comprehensive Meta-Analysis and a Network Medicine Observation

**DOI:** 10.3389/fnins.2021.606926

**Published:** 2021-02-24

**Authors:** Jiayu Shen, Yuan Hou, Yadi Zhou, Reena Mehra, Lara Jehi, Feixiong Cheng

**Affiliations:** ^1^Genomic Medicine Institute, Lerner Research Institute, Cleveland Clinic, Cleveland, OH, United States; ^2^Neurological Institute, Cleveland Clinic, Cleveland, OH, United States; ^3^Department of Molecular Medicine, Cleveland Clinic Lerner College of Medicine, Case Western Reserve University, Cleveland, OH, United States; ^4^Case Comprehensive Cancer Center, Case Western Reserve University School of Medicine, Cleveland, OH, United States

**Keywords:** coronavirus disease 2019 (COVID-19), cerebrovascular disease, protein-protein interactome, network medicine, SARS-CoV-2, stroke, vascular cell adhesion molecule 1 (VCAM-1)

## Abstract

The clinical characteristics and biological effects on the nervous system of infection with the severe acute respiratory syndrome coronavirus 2 (SARS-CoV-2) remain poorly understood. The aim of this study is to advance epidemiological and mechanistic understanding of the neurological manifestations of coronavirus disease 2019 (COVID-19) using stroke as a case study. In this study, we performed a meta-analysis of clinical studies reporting stroke history, intensive inflammatory response, and procoagulant state C-reactive protein (CRP), Procalcitonin (PCT), and coagulation indicator (D-dimer) in patients with COVID-19. Via network-based analysis of SARS-CoV-2 host genes and stroke-associated genes in the human protein-protein interactome, we inspected the underlying inflammatory mechanisms between COVID-19 and stroke. Finally, we further verified the network-based findings using three RNA-sequencing datasets generated from SARS-CoV-2 infected populations. We found that the overall pooled prevalence of stroke history was 2.98% (95% CI, 1.89–4.68; *I*^2^=69.2%) in the COVID-19 population. Notably, the severe group had a higher prevalence of stroke (6.06%; 95% CI 3.80–9.52; *I*^2^ = 42.6%) compare to the non-severe group (1.1%, 95% CI 0.72–1.71; *I*^2^ = 0.0%). There were increased levels of CRP, PCT, and D-dimer in severe illness, and the pooled mean difference was 40.7 mg/L (95% CI, 24.3–57.1), 0.07 μg/L (95% CI, 0.04–0.10) and 0.63 mg/L (95% CI, 0.28–0.97), respectively. Vascular cell adhesion molecule 1 (VCAM-1), one of the leukocyte adhesion molecules, is suspected to play a vital role of SARS-CoV-2 mediated inflammatory responses. RNA-sequencing data analyses of the SARS-CoV-2 infected patients further revealed the relative importance of inflammatory responses in COVID-19-associated neurological manifestations. In summary, we identified an elevated vulnerability of those with a history of stroke to severe COVID-19 underlying inflammatory responses (i.e., VCAM-1) and procoagulant pathways, suggesting monotonic relationships, thus implicating causality.

## Introduction

The ongoing global Coronavirus Disease 2019 (COVID-19), which is caused by the Severe Acute Respiratory Syndrome Coronavirus 2 (SARS-CoV-2), has led to more than 20 million confirmed cases and 700,000 deaths worldwide as of August 10, 2020 (Huang et al., 2020). The United States alone has 5 million confirmed cases with over 160,000 deaths, with dire, unprecedented social and economic consequences (Wu et al., 2020). Specifically, SARS-CoV-2 is known to cause substantial pulmonary disease, including pneumonia and acute respiratory distress syndrome (ARDS). The incidence of cerebrovascular disease among patients with COVID-19 is estimated to be ∼2% (Mao et al., 2020). Yet, the full spectrum of the neurological manifestations related to COVID-19 remains very poorly defined. For example, a recent study suggest that cerebrovascular disease could even happen in younger COVID-19 patients without traditional cardiovascular risk factors (Oxley et al., 2020). Previous studies have also reported stroke history was an independent risk factor associated with fatal outcome in patients with COVID-19. While limited by the small sample size and high heterogeneity among studies, the prevalence of stroke history in severe patients seems to be higher than that in non-severe patients (Chen et al., 2020). Several pathophysiological pathways related to cytokine release and secondary endothelial damage have been proposed to link SARS-CoV-2 infection with a hypercoagulable state (Ciceri et al., 2020). And the wide expression of angiotensin-converting enzyme 2 (ACE2), the functional receptor that mediates entry of SARS-CoV-2 into host cells with the help of co-expressed TMPRSS2, Furin and Neuropilin-1 (Nrp1) on the cell surface (Cantuti-Castelvetri et al., 2020; Hoffmann et al., 2020; Hou et al., 2020; Walls et al., 2020), constitutes a potential target in the endothelium of brain vessels (Varga et al., 2020). In addition, the depletion of ACE2 may have pro-inflammatory and vasoconstrictive effects (Hess et al., 2020). Until now, however, the underlying mechanisms of stroke in COVID-19 patients remain poorly understood, which requires a new approach to explore the underlying inflammatory endophenotypes between COVID-19 and stroke.

Recent network medicine studies suggested that proteins/genes that associate with and functionally govern a disease (i.e., COVID-19) phenotypes are localized in the corresponding disease module or subnetwork within the human protein-protein interaction (PPI) network as demonstrated in recent studies (Cheng et al., 2018, 2019a; Zhou et al., 2020b,c). Within the complex intracellular network, a disease is more likely to be the comprehensive effect of the interaction of multiple genes, rather than the consequence of an abnormality in a single gene. A better understanding of the implications of cellular interconnectedness (i.e., PPIs) on disease pathobiology/physiology could lead to identification of more accurate diagnosis and offer better targets for personalized treatment (Cheng et al., 2018, 2019a; Zhou et al.,2020a,b).

In this study, we performed a meta-analysis of clinical studies reporting stroke history, intensive inflammatory response and procoagulant state in patients with COVID-19. With the state-of-the-art network medicine-methodologies, we aim to inspect the underlying inflammatory endophenotypes between stroke and COVID-19 by incorporating disease-associated genes and SARS-CoV-2 host factors under the human interactome network model.

## Materials and Methods

All data used in this study are available in Supplementary Tables S1–S8 and all analytic codes are available from the corresponding author upon reasonable request.

### Search Strategy and Selection Criteria

As Supplementary Figure S1 shows, a search was conducted in PubMed and Embase on April 25th, 2020, using the keywords “COVID-19” or “SARS-CoV-2” and “clinical characteristics” or “clinical outcome” or “stroke” or “cerebrovascular disorder” or “cerebrovascular disease” or “C-reactive protein” or “Procalcitonin” or “D-dimer.” We excluded the following studies: reviews; case reports; letters; comments; editorials; and studies pertaining specific populations, such as pregnancy and children. Our team independently assessed eligibility of the included records and the data were subsequently extracted based on the following variables: author, study period, sample size, disease severity, age, comorbidity of stroke, serum levels of C-reactive protein (CRP), Procalcitonin (PCT), and D-dimer. A clinically definition of disease severity (i.e., patients requiring mechanical ventilation, vital life support, intensive care unit admission, death) was defined according to the American Thoracic Society guidelines for community-acquired pneumonia (Metlay et al., 2019).

### Data Extraction and Statistical Analysis

Two authors independently assessed for eligibility of the included records and the data were subsequently extracted by the same authors. Any disagreement was resolved by consensus and discussion. A meta-analysis was performed to estimate the pooled prevalence of stroke history in three COVID-19 patient groups (overall, severe, and non-severe), respectively. Random intercept logistic regression model was used to estimate pooled prevalence, with the Maximum-Likelihood Estimator to qualify the heterogeneity of studies. The continuous outcome data including CRP, PCT, and D-dimer, were transformed to mean and standard deviations from a previous approach (Wan et al., 2014) based on sample size, median and interquartile range. Next, we used inverse variance method to estimate the pooled mean difference (MD), with the DerSimonian-Laird method to estimate the variance among studies. The *tau*^2^ and *I*^2^ statistic were calculated to quantify the heterogeneity among studies in our meta-analysis. We considered *I*^2^ ≤ 50% as low heterogeneity among studies, 50% < *I*^2^≤ 75% as moderate heterogeneity and *I*^2^> 75% as high heterogeneity. All meta-analyses were conducted by meta and dmetar packages in R 3.6.3 platform^1^.

### Collection of Disease-Associated Genes for Stroke and COVID-19

Thirty-seven stroke-associated genes were extracted from the Human Gene Mutation Database (HGMD) (Stenson et al., 2017). A text search was performed using “stroke” as the keyword. Resulting disease terms from HGMD were verified for their relevance with stroke. For COVID-19, we used a combination of two gene sets: (i) a pan-coronavirus-host interactome from our recent study (Zhou et al., 2020a) containing the key human proteins involved in the infection of several coronaviruses based on literature evidence; and (ii) a SARS-CoV-2 viral protein-human protein interactome (Gordon et al., 2020) identified by affinity purification-mass spectrometry. The final COVID-19 target gene list contains 460 human genes.

### Building the Human Protein-Protein Interactome

The human protein-protein interactome containing 351,444 unique protein-protein interactions (PPIs, edges) connecting 17,706 proteins (nodes) used as the basis for the network analysis was based on our previous study (Cheng et al., 2018, 2019b). Briefly, a total of 18 bioinformatics and systems biology databases with five types of experimental evidences were used to build the comprehensive human interactome: (i) Binary PPIs tested by high-throughput yeast-two-hybrid (Y2H) systems; (ii) Binary, physical PPIs from protein three-dimensional (3D) structures; (iii) Signaling network by literature-derived low-throughput experiments; (iv) Kinase-substrate interactions by literature-derived low-throughput or high-throughput experiments;, and (v) Literature-curated PPIs identified by affinity purification followed by mass spectrometry (AP-MS), Y2H, or by literature-derived low-throughput experiments. All inferred data, including evolutionary analysis, gene expression data, and metabolic associations, were excluded. The genes were mapped to their Entrez ID based on the NCBI database (Sayers et al., 2019). Gene symbols were based on GeneCards^2^. Detailed descriptions for building the human protein-protein interactome are provided in our previous studies (Cheng et al., 2018, 2019b).

### Network Proximity Measure

We quantified the network proximity of stroke and COVID-19 in the human interactome using the “closest” measure (Cheng et al., 2019a) for stroke genes *S* and COVID-19 genes *C*:

$$\left\langle d_{S\,C}\right\rangle =\frac{1}{∥S∥+∥C∥}\,\left(\sum_{s\in S}m\,i\,n_{c\in C}\,d\,\left(s,c\right)+\sum_{c\in C}m\,i\,n_{s\in S}\,d\,\left(s,c\right)\right)$$

where *d*(*s*,*c*) is the shortest distance of a stroke gene *s* and a COVID-19 gene *c* in the human protein-protein interactome. A permutation test of 1,000 repeats was performed to estimate the average proximities of two random gene lists with similar degree distributions to those of *S* and *C*. The Z score was then calculated as:

$$Z_{d_{S\,C}}=\frac{d_{S\,C}-\bar{d_{r}}}{\sigma_{r}}$$

where $\bar{d_{r}}$ and σ_*r*_ were the mean and standard deviation of the permutation test. *P*-value was calculated based on the permutation test. *P* < 0.05 was considered statistically significant. Network visualization and subnetwork (modularity) analyses were performed using Cytoscape (v.3.8.2)^3^.

### Functional Enrichment Analysis

Functional enrichment analyses for the network genes were performed using Enrichr (Kuleshov et al., 2016).

### Gene Expression Analysis Using SARS-CoV-2-Infected Populations

In this study, we used three RNA-sequencing datasets of COVID-19 patients vs. controls. All RNA-sequencing datasets were retrieved from the National Center of Biotechnology Information (NCBI) Gene Expression Omnibus (GEO) database^4^. (1) Nasal samples (GEO ID: GSE152075) (Lieberman et al., 2020) were collected from 430 individuals with SARS-CoV-2 and 54 controls; (2) Peripheral blood mononuclear cell (PBMC) samples (GEO ID: GSE157103) (Overmyer et al., 2020) collected from 99 COVID-19 positive patients and 26 controls; and (3) induced pluripotent stem cells (iPSC)–cardiomyocytes (GSE150392) (Sharma et al., 2020) from 3 SARS-CoV-2 infected iPSC-cardiomyocytes vs. mock control. The analyses of differential expression genes were performed by edgeR 3.12 (McCarthy et al., 2012) in the R 4.0.3 platform. To eliminate the influence from covariates, gene differential expression analyses were adjusted for age and sex. Adjusted *p*-value (q) were computed by Benjamini-Hochberg method (Benjamini and Hochberg, 1995). We defined significantly differentially expressed genes using | log_2_-fold change| > 0.5 and adjusted *p*-value [*q*] < 0.05.

## Results

### Meta-Analysis for COVID-19 Patients With a History of Stroke

In this study, a total of 1,054 unique publications of COVID-19 were identified after being deduplicated, of which 115 studies were assessed via title and abstract. 12 studies (*n* = 2,509 patients) reporting stroke in a COVID-19 positive-tested population were eligible for meta-analysis. Among them, 9 studies divided the included patients (*n* = 2,371) into severe and non-severe groups. The flow chart of study selection and essential characteristics of studies is shown in Supplementary Figure S1.

**FIGURE 1:**
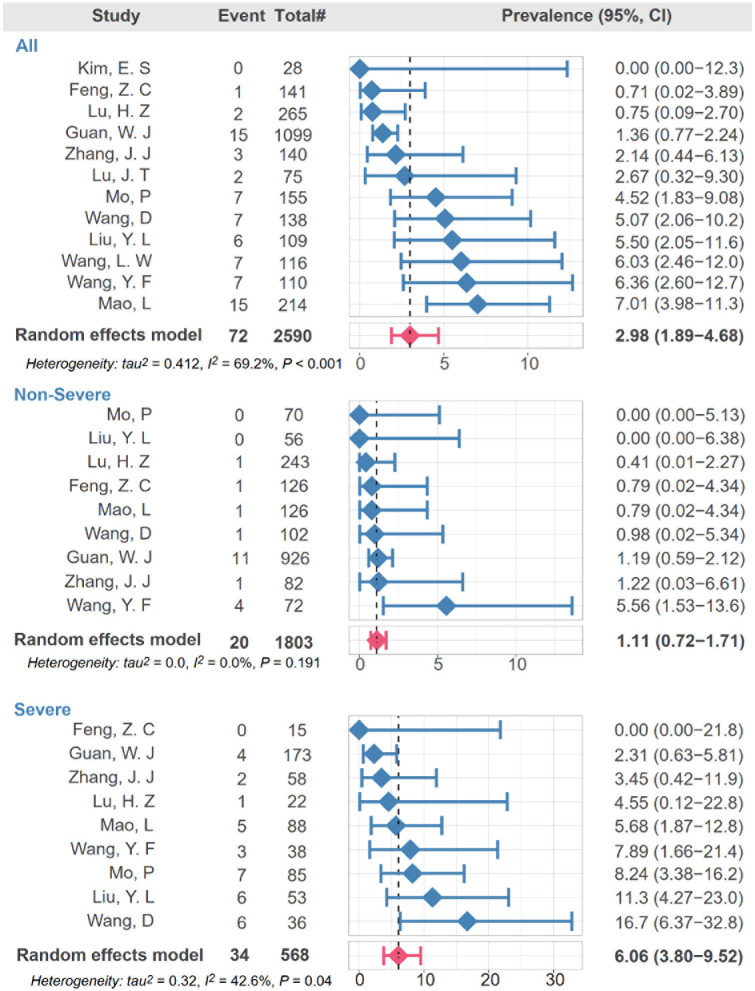
Meta-analysis revealing association of stroke with disease severity of COVID-19. Random intercept logistic regression model was used to estimate pooled prevalence and *I*^2^ was used to show heterogeneity among studies. The red bar denotes the pooled prevalence using random effect model.

In the overall COVID-19 population, the pooled prevalence of stroke history was 2.98% (95% CI, 1.89–4.68%) with moderate heterogeneity (*I*^2^ = 69.2%). While the prevalence of stroke history in patients with severe COVID-19 illness was significantly higher (6.06%; 95% CI, 3.8–9.52) than that in the non-severe group (1.11%; 95% CI, 0.72–1.71%) (Figure 1). The heterogeneity among the studies in severe and non-severe group were low, with *I*^2^ statistics of 0.0 and 42.6%, respectively.

**FIGURE 2:**
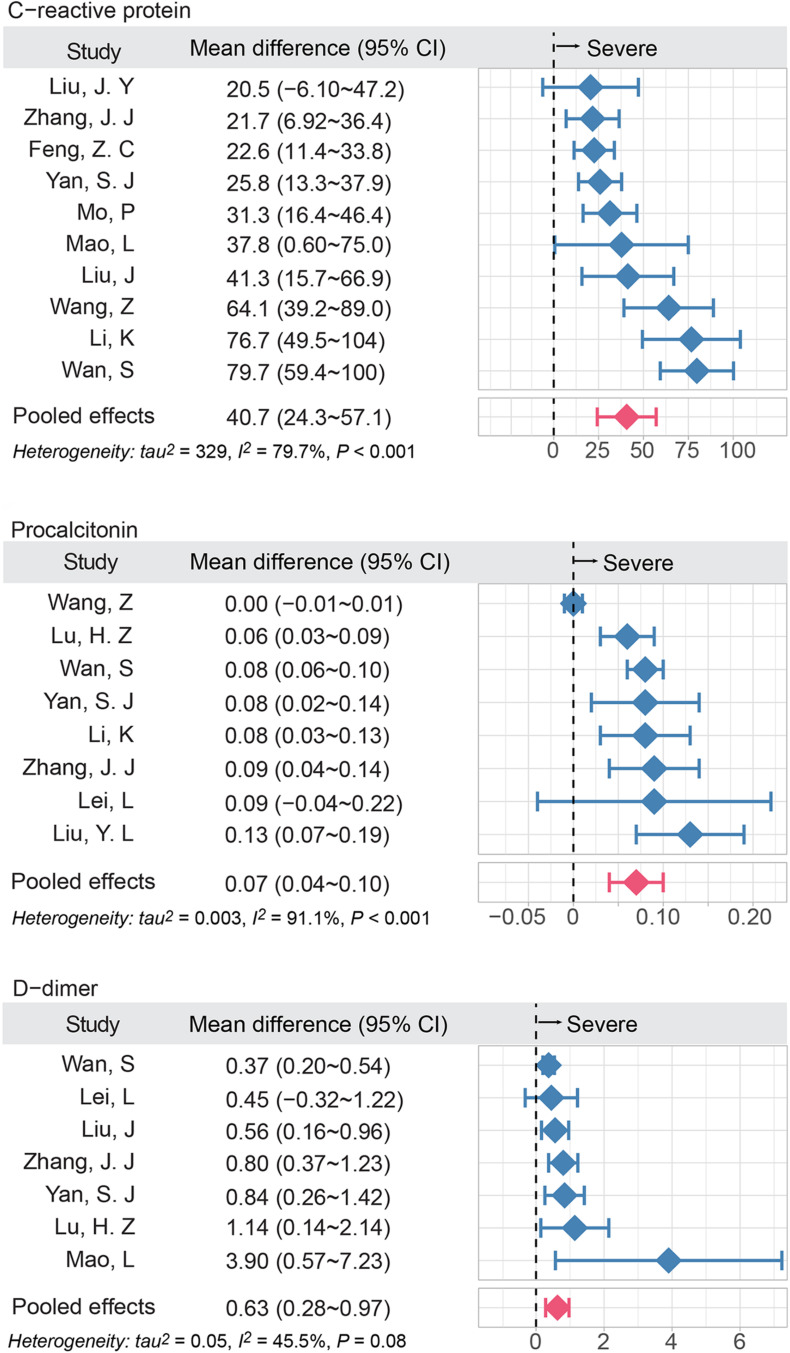
The elevated inflammatory factors and coagulation indicator are associated with disease severity of COVID-19. The meta-analysis (inverse variance method) was performed to estimate the MD in three groups. *I*^2^ was used to show heterogeneity among studies. The red bar denoted the pooled MD using random effect model; MD, mean difference.

In terms of the results of laboratory examinations, two inflammatory factors, CRP and PCT, were significantly higher in the severe group compared to the non-severe group. The mean difference changes of CRP and PCT were 40.7 mg/L (95% CI, 24.3–57.1; *I*^2^ = 79.7%) and 0.07 μg/L (95% CI, 0.04–0.10; *I*^2^ = 91.1%), respectively. The procoagulant indicator, D-dimer, was higher in the severe group, compared to the non-severe group (MD = 0.63 mg/L; 95%CI, 0.28–0.97; *I*^2^ = 45.5%) (Figure 2).

### Discovery of Inflammatory Endophenotypes by Network Analysis

**FIGURE 3:**
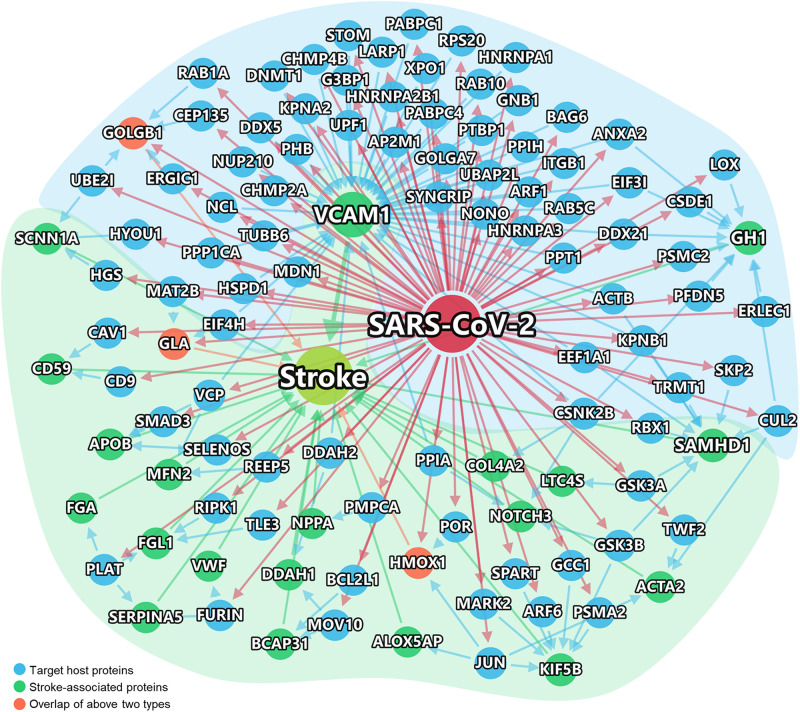
Network-inferred inflammatory endophenotypes shared by stroke and COVID-19. Virus target genes are shown in Blue. The stroke-associated genes were shown in Green. Stroke-associated genes which were also the direct targets of the virus, shown in Orange. The links between genes/proteins denote the physical protein-protein interactions (including SARS-CoV-2 viral protein and human protein interactions, and human protein-protein interactions).

Recent studies have suggested that different diseases often have common underlying mechanisms and shared intermediate pathophenotypes, or endo(pheno)types (Ghiassian et al., 2016). For example, inflammatory endophenotypes has been identified in a variety of human diseases, including cardiovascular disease (Ghiassian et al., 2016) and COVID-19 (Zhou et al., 2020b). We next turned to inspect the inflammatory endophenotypes between stroke and COVID-19 by incorporating SARS-CoV-2 host genes and the stroke-associated genes using network-based analysis. Using network proximity analysis from the human PPIs (Cheng et al., 2018; Zhou et al., 2020b), we found that there were multiple key PPIs (blue arrows) between the SARS-CoV-2 host genes (blue nodes) and stroke-associated genes (green nodes) (*Z* = −1.9, *P* = 0.019 [permutation test]) (Figure 3). Three genes (orange), golgin subfamily B member 1 (GOLGB1), α-galactosidase A (GLA), and heme oxygenase-1 (HMOX1), are both SARS-CoV-2 host genes as well as stroke-associated genes. The inflammatory endophenotype network between stroke and COVID-19 is composed of three subnetworks (Supplementary Figure S3), in which the largest one is centered around the hub gene *VCAM-1*. We further performed functional enrichment to identify possible pathways between SARS-CoV-2 infection and stroke. As shown in Supplementary Figures S4–S7, we found that several enriched pathways and gene ontology terms, such as endocytosis, NF-kappa B signaling pathway, viral life cycle, and cytokine-mediated signaling pathway (Supplementary Figure S4), offered possible mechanisms of comorbidities/complications of SARS-CoV-2 infection and stroke. The largest subnetwork (Supplementary Figure S3A) showed more significantly enriched pathways and gene ontology terms related to the viral life cycle or immune system responses (Supplementary Figure S5).

Among the network genes, we selected eight of them (VACM-1, SAMHD-1, DDAH-1, HMOX1, LTC4S, ACTB, KPNA2, and JUN) for further analysis based on a combination of factors: (i) strength of the network-predicted associations (Supplementary Table S5); (ii) each gene’s | log-fold change| > 0.5 and *q* < 0.05 (Supplementary Figures S6–S8); (iii) genes interact with at least two stroke-associated genes or SARS-CoV-2 target host genes;, and (iv) literature-reported evidence associated with stroke.

**FIGURE 4:**
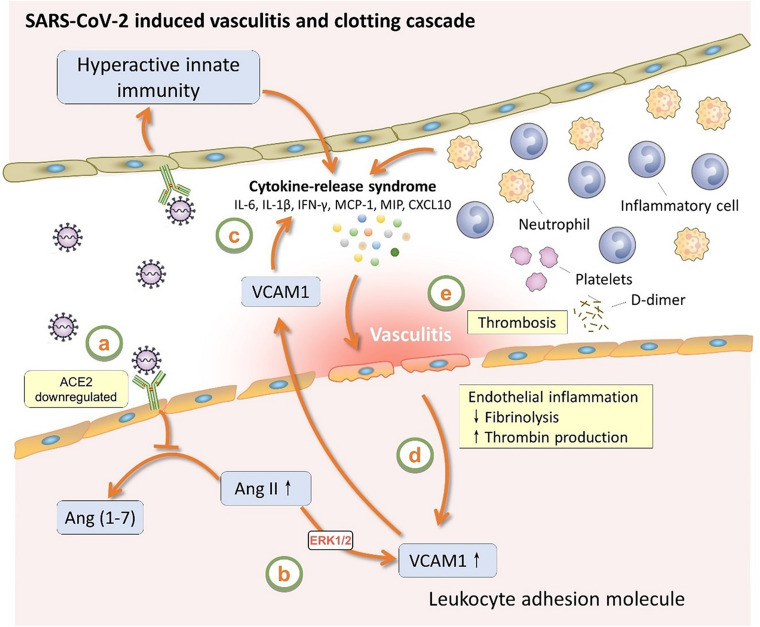
The role of VCAM1 in SARS-CoV-2 induced vasculitis and clotting cascade. VCAM1, vascular cell adhesion molecule 1; ACE2, angiotensin-converting enzyme 2; Ang II, angiotensin II; Ang (1–7), angiotensin (1–7); ERK1/2, extracellular signal-regulated kinase 1/2; IL-6, interleukin-6; IL-1β, interleukin-1β; IFN-γ, interferon-γ; MCP-1, monocyte chemoattractant protein-1; MIP, macrophage inflammatory protein.

As shown in Figure 3, VCAM-1 have the highest degree (number of PPIs) according to the PPI network analysis. Multiple host genes mediate the association of vascular cell adhesion molecule (VCAM)-1 with SARS-CoV-2, such as peptidyl-prolyl isomerase H (PPIH), charged multivesicular body protein 2B (CHMP2B), and nucleolin (NCL). A metabolomic data analysis had also confirmed the up-regulation of VCAM-1 in patient with COVID-19 (Shen et al., 2020). As shown in Figure 4, as a potential mechanism of downregulation of ACE2 related to SARS-CoV-2 cell entry process (Verdecchia et al., 2020; Zhang et al., 2020), the dysregulation of the renin-angiotensin-aldosterone system (RAAS) leads decreased cleavage of angiotensin II, which further stimulates the transcription of VCAM-1, one of important leukocyte adhesion molecules, through extracellular signal-regulated kinase (ERK) 1/2 signaling pathway (Montezano et al., 2010). The up-regulated VCAM-1 facilitates the accumulation and extravasation of neutrophil and inflammatory cells, and subsequently increases levels of interleukin (IL)-6, IL-1β, IFN-γ, monocyte chemoattractant protein-1 (MCP-1), macrophage inflammatory protein (MIP) and IP10 (CXCL10) (Huang et al., 2020). These activated leukocytes and proinflammatory cytokines may enhance vasculitis and disrupt endothelial function and integrity, leading to the further release of VCAM-1. More importantly, the disruption of vascular integrity also leads to the exposure of the thrombogenic basement membrane which facilitates the accumulation of platelets and D-dimer, and activation of the clotting cascade and eventually results in the thrombosis (Teuwen et al., 2020).

Meanwhile, other stroke-associated genes, such as SAM and HD domain containing deoxynucleoside triphosphate triphosphohydrolase-1 (SAMHD-1), dimethylarginine dimethylaminohydrolase-1 (DDAH-1), heme oxygenase 1 (HMOX1), leukotriene C4 synthase (LTC4S), actin beta (ACTB), and karyopherin subunit alpha 2 (KPNA2) and Jun proto-oncogene (JUN), which are potentially or directly targeted by SARS-CoV-2 may also play roles in inflammatory response and coagulopathy. For example, based on the fact that innate and adaptive immune processes are involved in medium- and large-vessel vasculitis (Weyand and Goronzy, 2003), SAMHD1, which is known to act as an immunomodulator in different viral infections and proinflammatory responses (Rice et al., 2009), is thought to be associated with cerebral vasculopathy and early onset stroke (Xin et al., 2011). DDAH-1, one of the main components involving in the pathway for asymmetric dimethylarginine (ADMA) clearance, has been realized to be the key regulator associated with thrombosis stroke or other vascular disease (Yoo and Lee, 2001; Leiper et al., 2007). Numerous studies have reported the effectiveness of HMOX1, a stress-responsive protein induced by various oxidative agents, in cardiology by aspects such as inflammation, antioxidant function, apoptosis, hypoxia, and ischemia/reperfusion injury (Wan et al., 2020). The upregulation of HMOX1 during cerebral ischemia revealed a protective effect on neuronal cell against oxidative stress (Nitti et al., 2018; Cui et al., 2020). A previous study also showed that the dysfunction or downregulation of LTC4S, one of the key enzymes of the 5-lipoxygenase pathway, was associated with increased risk of venous thromboembolism and ischemic stroke (Freiberg et al., 2010). Meanwhile, SARS-CoV-2 host genes, such as ACTB, KPNA2, and JUN, interact with SAMHD1, VCAM1, and HMOX1, in the PPI network, indicating possible mechanisms of COVID-19 associated stroke.

**FIGURE 5:**
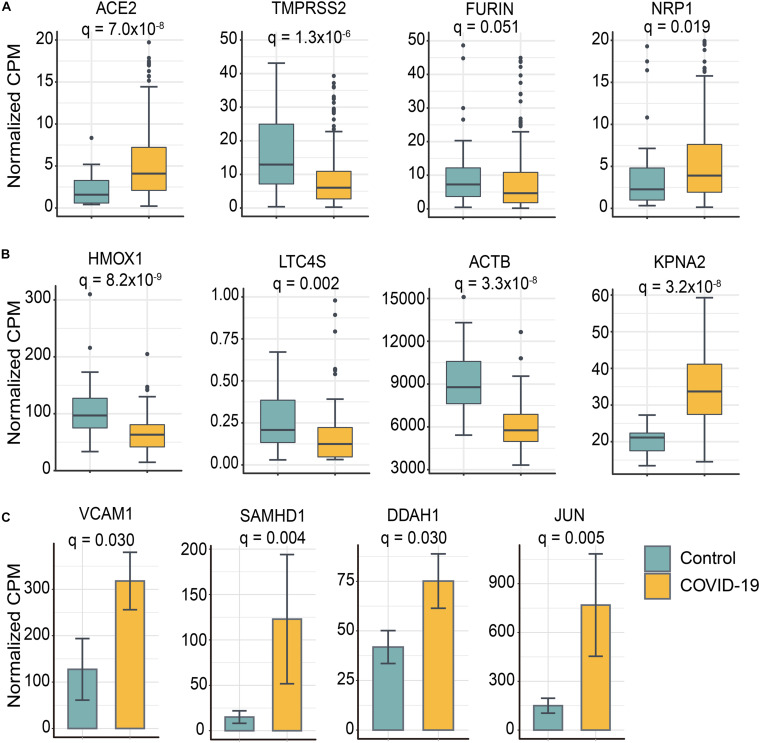
Selected genes involved in COVID-19-associated stroke were differentially expressed between SARS-CoV-2 positive patients and controls. **(A)** Four SARS-CoV-2 entry host factors were significantly differentially expressed in nasal tissues from COVID-19 positive patients compared to controls. **(B)** Stroke-related genes were differentially expressed in peripheral blood mononuclear cell (PBMC) samples from COVID-19 positive patients and controls. The data are represented as a boxplot where the middle line is the median, the lower and upper edges of the box are the first and third quartiles, the whiskers represent the interquartile range (IQR) × 1.5. **(C)** The bar plot shows the stroke-related genes were upregulated in iPSC–cardiomyocytes after SARS-CoV-2 infection. Adjusted *p*-value (q) were computed by Benjamini-Hochberg method. The cutoff of differentially expressed genes were | log_2_-fold change| > 0.5 and adjusted *p*-value (*q*) < 0.05 (Supplementary Tables S5–S8).

In order to further understand the underlying mechanisms of PPI network-based findings, we investigated the expression levels of the 8 selected stroke-associated genes (VACM-1, SAMHD-1, DDAH-1, HMOX1, LTC4S, ACTB, KPNA2, and JUN) and 4 SARS-CoV-2 host factors/genes (ACE2, TMPRSS2, Furin, and NRP1) between COVID-19 positive patients and negative individuals, respectively (Figure 5). Interestingly, except for FURIN, the expression level of ACE2, TMPRSS2, and NRP1 were significantly dysregulated in nasal tissues from COVID-19 positive patients. Moreover, the expression level of several stroke-associated inflammatory genes, such as VCAM-1, SMAHD-1, and DDAH1, were significantly upregulated in COVID-19 positive patients, indicating that the inflammatory response may play potential roles in mediating COVID-19-associated stroke. As mentioned above, the expression levels of HMOX1, LTC4S, and ACTB seem to have negative correlation relationships with the risk of cerebral ischemia (Freiberg et al., 2010; Nitti et al., 2018; Cui et al., 2020; Yang et al., 2020), the significant downregulation of HMOX1, LTC4S, and ACTB in PBMC samples from COVID-19 positive patients may indicate possible molecular mechanisms between COVID-19 and stroke. However, further clinical and functional studies using COVID-19 patient samples or animal models are highly needed to investigate direct viral infection effects and indirect inflammatory effects on human brains by SARS-CoV-2.

## Discussion

There is growing evidence of neurological manifestations of COVID-19 (Zubair et al., 2020). Acute stroke of varying arterial and venous mechanisms (in up to 6% of those with severe illness; Yaghi et al., 2020) is one of the more-severe presentations of COVID-19, associated with worsen prognosis. And the pooled prevalence of the history of stroke in patients with severe COVID-19 was estimated to be 2.98% in our study, consistent with previous studies (Chen et al., 2020). We adopted the network medicine-based approaches to investigate the stroke-associated genes, especially VCAM-1, involved in the comorbidities between stroke and COVID-19 and preliminarily inspect the underlying inflammatory endophenotypes between COVID-19 and stroke.

According to the latest results of epidemiological investigation, the approximate prevalence of stroke was 1.1% in China, bearing the biggest stroke burden in the world (Wang et al., 2017). Over the course of the COVID-19 pandemic, people with stroke history were reported to have worse outcomes in COVID-19 positive population (Qin et al., 2020). Even though the overall prevalence of stroke was threefold higher than that in general population (2.98%) (Wang et al., 2017), it was interesting to note the stroke prevalence (1.11%, Figure 1) in the non-severe group was similar with that of general population, and lower than that in severe group (6.06%, Figure 1). Recent studies also reported patients with COVID-19 initially presented with stroke and the incidence of stroke was reported to be 5.7% in patients with severe illness, compared with 0.8% in those with non-severe illness (Mao et al., 2020). An autopsy series of 27 patients with COVID-19 detected SARS-CoV-2 in multiple extra pulmonary organs, and the organotropism of SARS-CoV-2 may influence the course of COVID-19 and possibly aggravate preexisting conditions (Puelles et al., 2020). Meanwhile, the intensive inflammatory response and procoagulant status in COVID-19 patients may contribute to the incidence of stroke (Sellner et al., 2020). Consistent with previous studies, the levels of CRP, PCT, and D-dimer in our study were higher in severe group compared to those in non-severe group (Figure 2). However, the underlying pathobiology of COVID-19-related stroke remains unknown.

Direct viral toxicity, endothelial cell damage, and thromboinflammation, dysregulation of the immune response and dysregulation of the RAAS are now suspected to be the key mechanisms mediating multiorgan dysfunction in COVID-19 patients (Gupta et al., 2020). Even though some studies have reported SARS-CoV-2 can be detected in cerebrospinal fluid and brain (2020; Puelles et al., 2020), other neurological manifestations of COVID-19 perhaps reflecting the proinflammatory and prothrombotic cascade in the wake of cytokine storm as it affects brain vasculature and the blood-brain barrier (Rice et al., 2009; Teuwen et al., 2020). Here we used PPI network analysis to screen some stroke-associated genes which interact with SARS-CoV-2 host genes. As presented in Figure 4, one stroke-associated gene, VCAM-1, connects the viral entry process, dysregulation of the RAAS and immune response, vessel endothelial cell damage, and clotting cascade together, indicating that we should recognize SARS-CoV-2 infection from a holistic perspective, rather than independent mechanism. Furthermore, the roles of other stroke-associated genes, such as SAMHD-1, DDAH-1, HMOX1, LTC4S, ACTB, KPNA2, and JUN, in mediating stroke secondary to SARS-CoV-2 infection are required to be further explored experimental using COVID-19 patient samples or SARS-CoV-2 animal models.

We acknowledge several limitations in our study. First, as most of the included studies are from China, the existence of susceptibility difference among different races may further contribute to the heterogeneity. There was also significant heterogeneity in estimating the levels of inflammatory factors due to the incomplete record of medication history and treatment approaches in the original publications. Moreover, the network proximity analysis without considering direction of PPIs can only show the potential associations and our knowledge of the human protein-protein interactome is not complete. Previous studies showed that PPI network analysis without directions had a good performance in identifying network-based disease-disease relationships (Menche et al., 2015), including COVID-19 (Zhou et al., 2020c). Integrative network analysis with directed networks, including transcriptional regulatory networks (protein-DNA interactions), are highly needed in the future. In addition, integration of more comprehensive SARS-CoV-2 interactome, such as SARS-CoV-2 virus-host PPIs and genetic interactions identified by CRISPR-Cas9 screenings (Daniloski et al., 2020), may improve performance of network proximity analysis further.

## Conclusion

In conclusion, our pooled analysis of existing data suggests potential dose-response relationships and support COVID-19-stroke causality and the human interactome analyses characterize candidate underlying inflammatory response and procoagulant pathways implicated in stroke and COVID-19. Findings translate into clinical relevance in terms of need for heightened clinical suspicion for stroke particularly in those with severe COVID-19.

## Data Availability Statement

The original contributions presented in the study are included in the article/Supplementary Material, further inquiries can be directed to the corresponding author.

## Author Contributions

FC conceived the study. JS, YH, and YZ performed all experiments and data analysis. LJ and RM discussed and interpreted results. FC, JS, YH, and YZ wrote and critically revised the manuscript with contributions from other co-authors. All authors contributed to the article and approved the submitted version.

## Conflict of Interest

The authors declare that the research was conducted in the absence of any commercial or financial relationships that could be construed as a potential conflict of interest.

## Footnotes

1 www.r-project.org

2 https://www.genecards.org/

3 https://cytoscape.org

4 https://www.ncbi.nlm.nih.gov/geo/
