# Supplementary material for: The Epidemiological and Mechanistic Understanding of the Neurological Manifestations of COVID-19: A Comprehensive Meta-Analysis and a Network Medicine Observation
Source: Front Neurosci. 2021 Feb 24;15:606926. doi: 10.3389/fnins.2021.606926 (PMC7959722; doi:10.3389/fnins.2021.606926)
Supplement: Supplementary file 1 [file Data_Sheet_1.pdf]

SUPPORTING INFORMATION

The epidemiological and mechanistic understanding of the neurological manifestations of COVID-19: a comprehensive meta-analysis and a network medicine observation

*Correspondence to: Feixiong Cheng, PhD

Lerner Research Institute, Cleveland Clinic

Tel: 216-4447654; Fax: 216-6360009

Email: chengf@ccf.org

Supplementary Figures

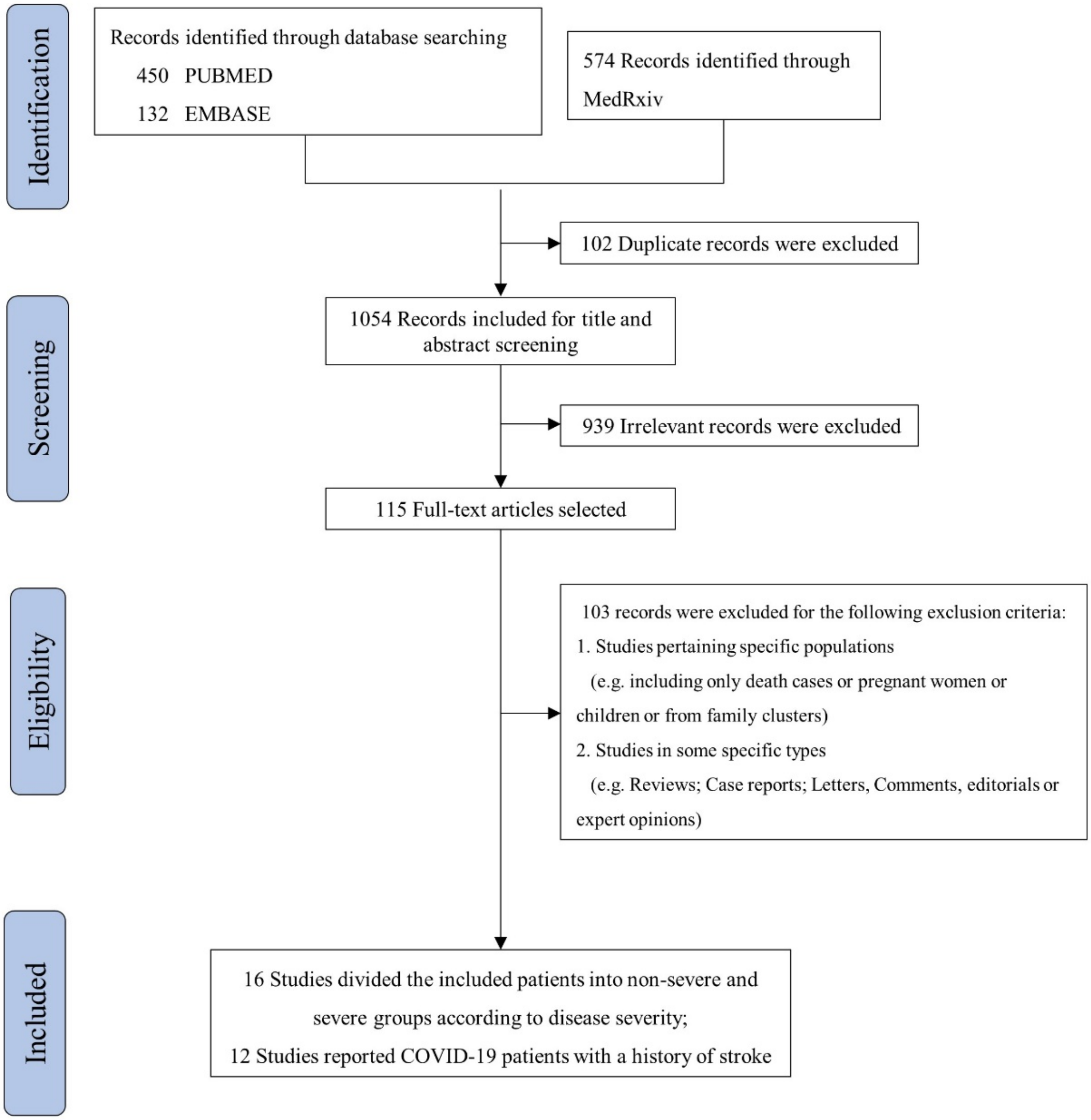

Figure S1. Systematic literature review process. The flow diagram describes the systematic review of the literature for the proportion of stroke/cerebrovascular disease in patients with COVID-19. Twelve studies were finally included for meta-analysis. Among them, nine studies divided their patients into non-severe group and severe group according to the illness severity.

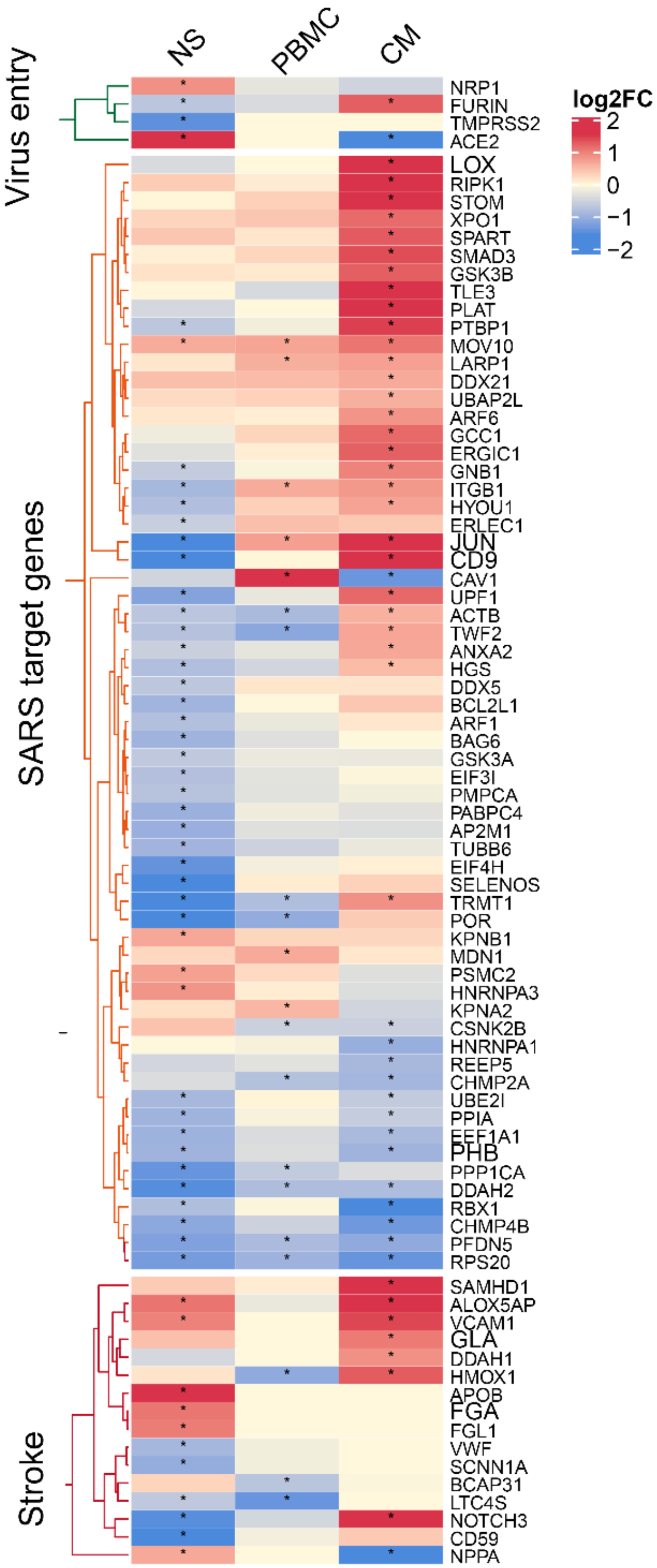

Figure S2. The expression levels of virus entry-associated genes, SARS-CoV-2 host genes, and stroke-associated genes in three RNA-seq datasets. NS, nasal samples; PBMC, peripheral blood mononuclear cell; CM, cardiomyocytes.

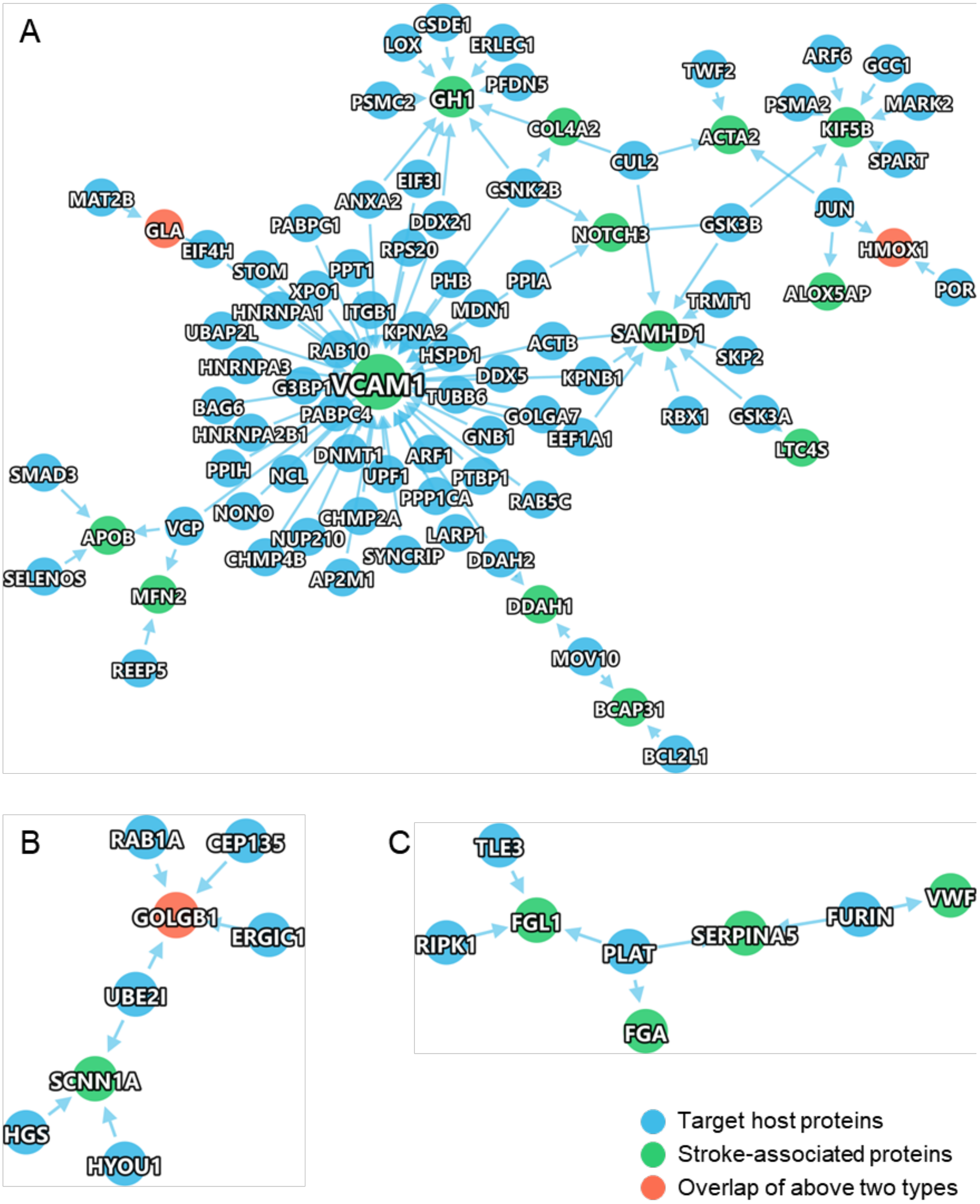

Figure S3. The three main subnetworks from the inflammatory endophenotype network shared by stroke and COVID-19.

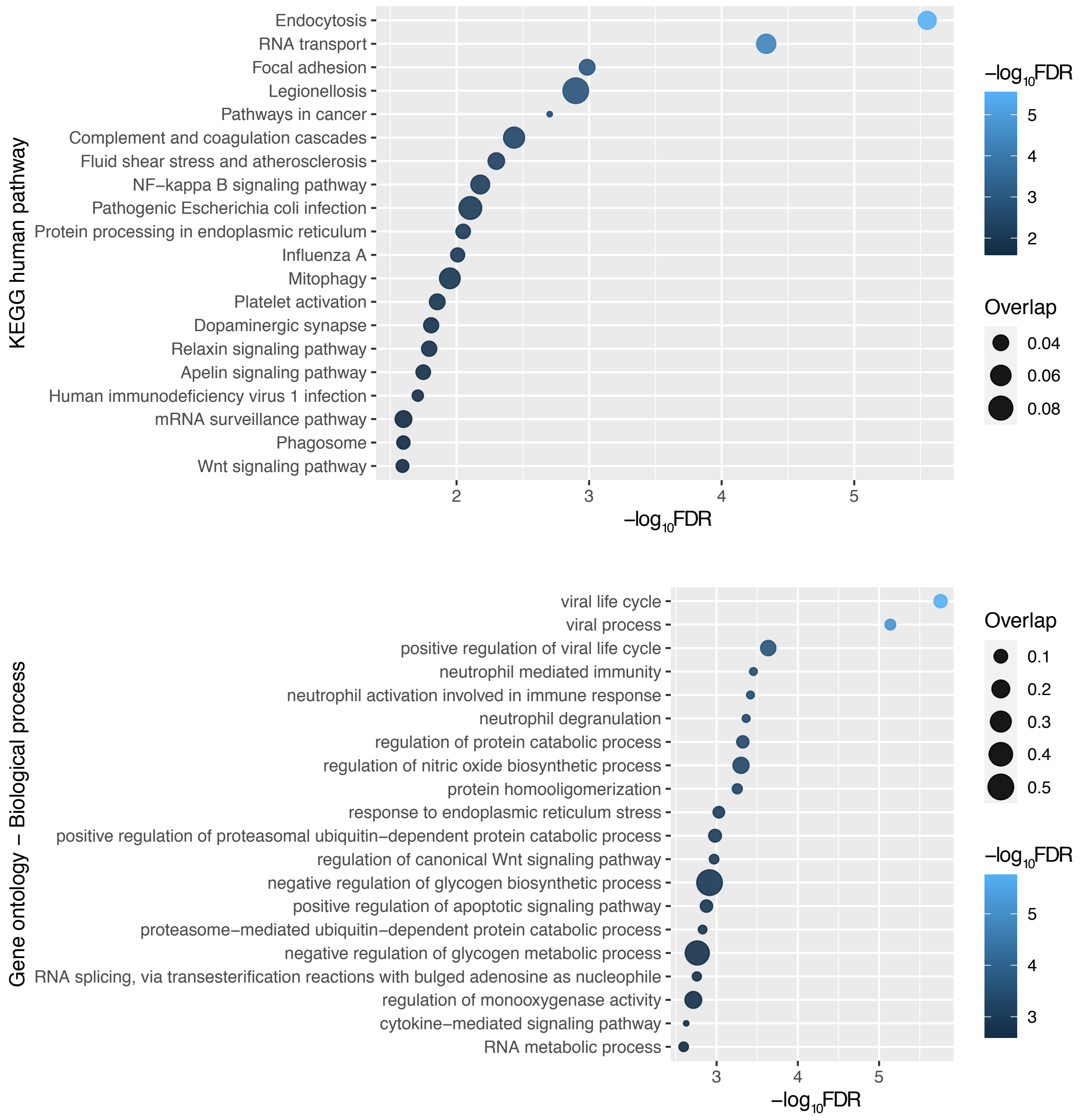

Figure S4. Functional enrichment analysis results for the network-inferred inflammatory endophenotypes shared by stroke and COVID-19.

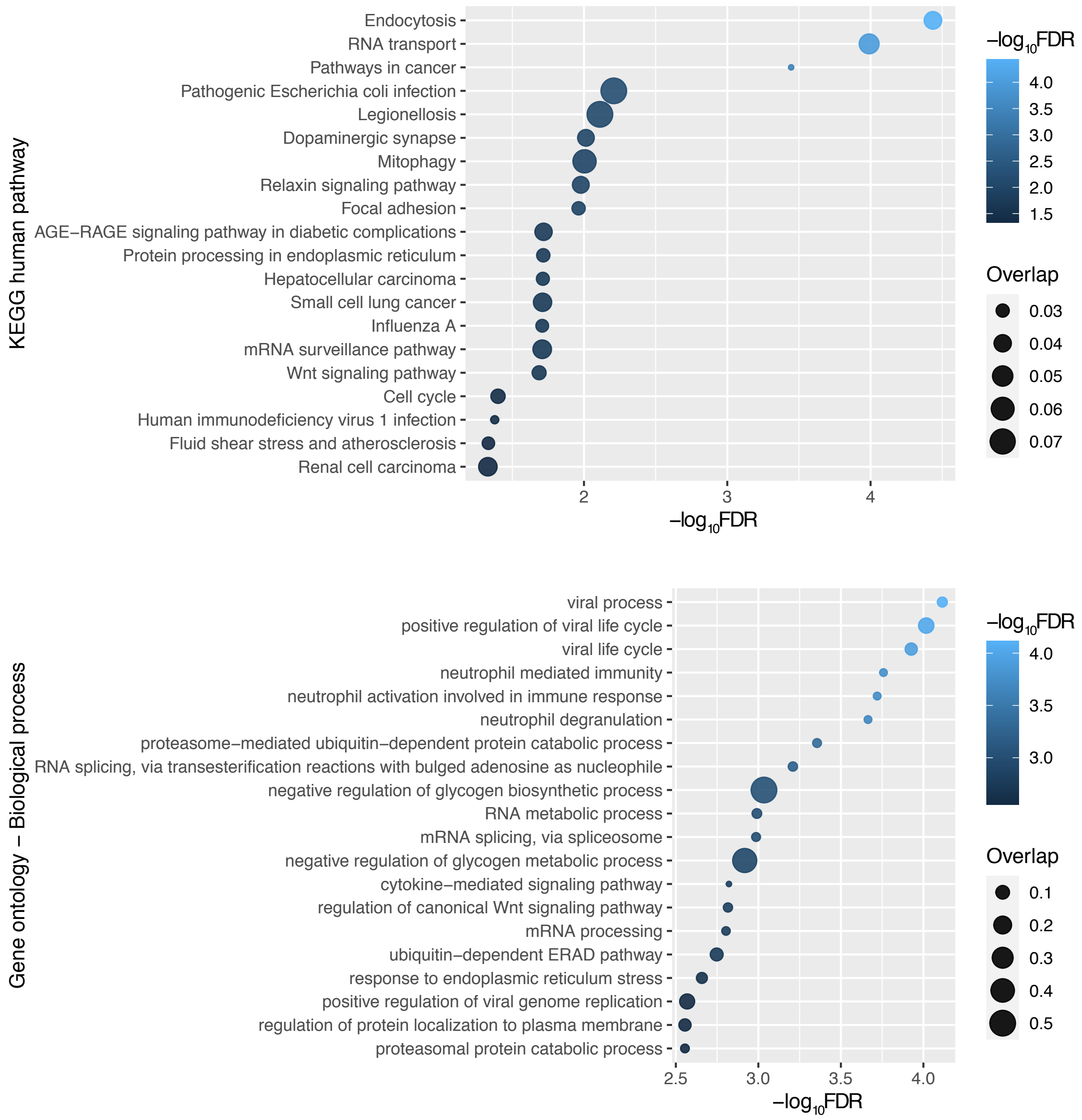

Figure S5. Functional enrichment analysis results for the subnetwork in Figure S3A.

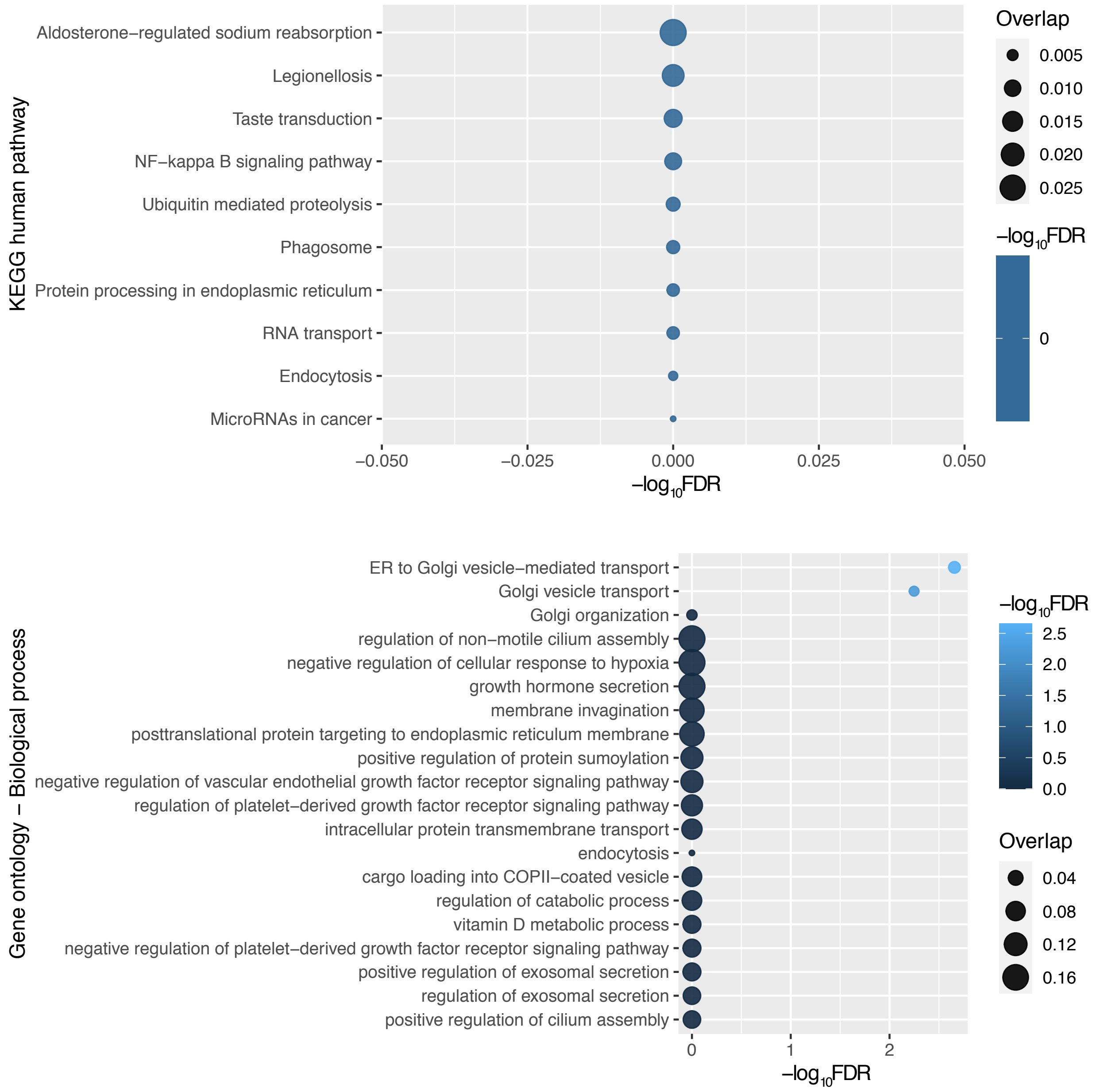

Figure S6. Functional enrichment analysis results for the subnetwork in Figure S3B.

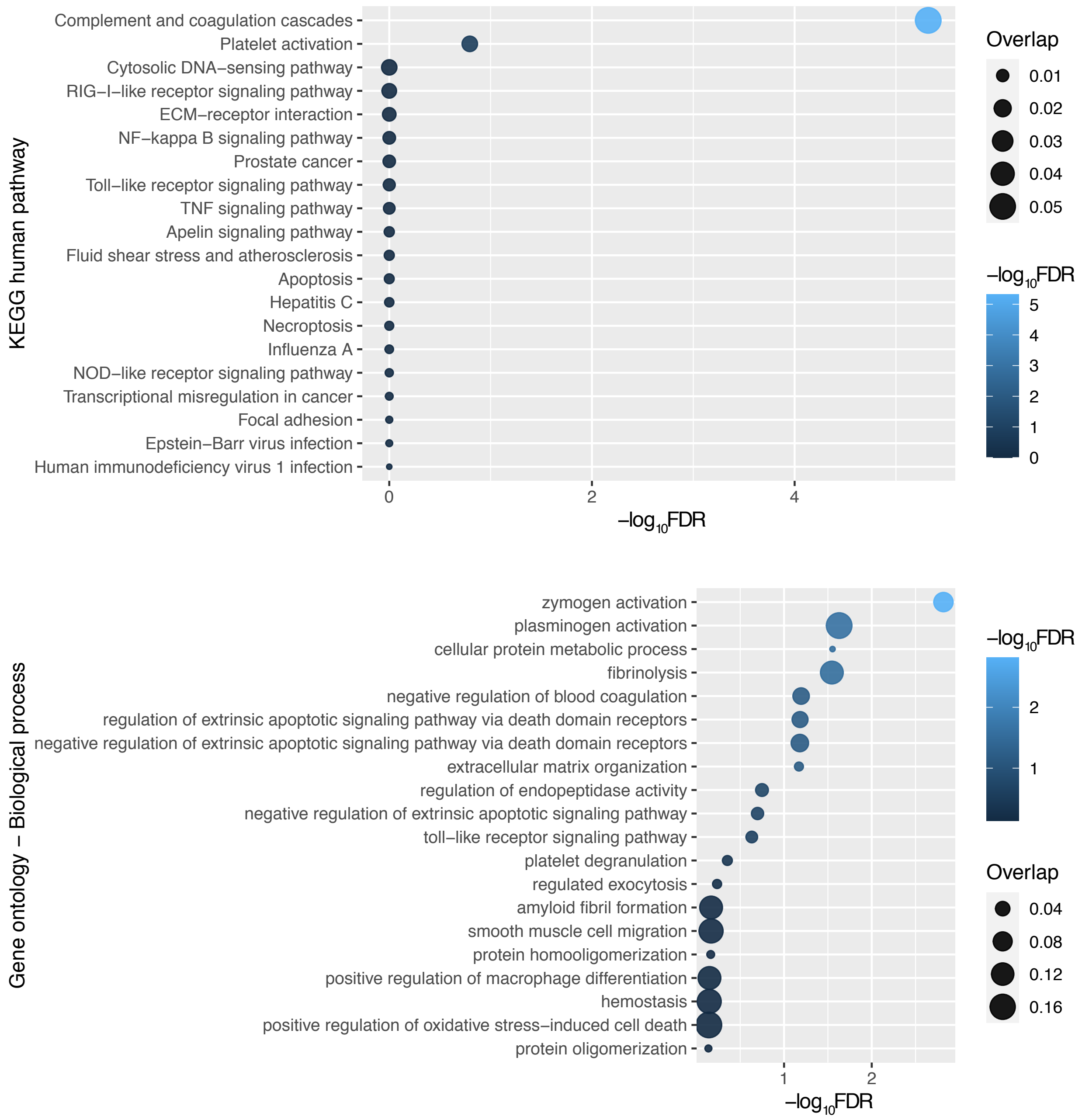

Figure S7. Functional enrichment analysis results for the subnetwork in Figure S3C.

Supplementary Tables

Table S1. Characteristics of patients with a history of stroke.

Studies	Date	Country	Sample size	Non-severe patients				Severe patients			
				n (%)	Age (yrs)[a]	Men (%)	Stroke, n (%)	n (%)	Age (yrs)[a]	Men (%)	Stroke, n (%)
J. J, Zhang et al[1]	Jan.16-Feb.3, 2020	China	140	82 (58.6)	51.5 (26-78)	38 (46.3)	1 (1.2)	58 (41.4)	64 (25-87)	33 (56.9)	2 (3.4)
P, Mo et al[2]	Jan. 1- Feb. 5, 2020	China	155	70 (45.2)	46 (35-56)	31 (44.3)	0 (0)	85 (54.8)	61 (51-70)	55 (64.7)	7 (8.2)
D, Wang et al[3]	Jan.1 - 28, 2020	China	138	102 (73.9)	51 (37-62)	53 (52)	1 (1)	36 (26.1)	66 (57-78)	22 (61.1)	6 (16.7)
Z. C, Feng et al[4]	Jan.17 - Feb.1, 2020	China	141	126 (89.4)	41 (33-52)	65 (51.6)	1 (0.8)	15 (10.6)	58 (44-66)	7 (46.7)	0 (0)
Y. L, Liu et al[5]	Jan.2 - Feb.1, 2020	China	109	56 (51.4)	49 (37-59)	31 (55.4)	0 (0)	53 (48.6)	61 (52-70)	28 (52.8)	6 (11.3)
H. Z, Lu et al[6]	Jan.1 - Feb.9, 2020	China	265	243 (91.7)	NA	NA	1 (0.4)	22 (8.3)	NA	NA	1 (4.5)
L, Mao et al[7]	Jan.16 - 19, 2020	China	214	126 (58.9)	48.9±14.7	43 （34.1）	1 (0.8)	88 (41.1)	58.2±15	44 （50）	5 (5.7)
Y. F, Wang et al[8]	Jan.1 - Feb.10, 2020	China	110	72 (65.5)	NA	24 (33.33)	4 (5.6)	38 (34.5)	NA	24 (63.16)	3 (7.9)
W. J, Guan et al[9]	Dec. 1- Jan. 29 2020	China	1099	926 (84.3)	45 (34-58)	537 (58.2)	11 (1.2)	173 (15.7)	52 (40-65)	100 (57.8)	4 (2.3)

[a] Data are presented as median (IQR) or mean (SD); NA, not available;

Table S2. C-reactive protein.

Studies	Date	Country	Sample size	Non-severe patients				Severe patients			
				n (%)	Age(yrs) [a]	Men (%)	CRP (mg/L) [b]	n (%)	Age (yrs) [a]	Men (%)	CRP (mg/L) [b]
J. J, Zhang et al[1]	Jan.16-Feb.3, 2020	China	140	82 (58.6)	51.5 (26-78)	38 (46.3)	28.7 (9.5-52.1)	58 (41.4)	64 (25-87)	33 (56.9)	47.6 (20.6-87.1)
P, Mo et al[2]	Jan. 1-Feb. 5, 2020	China	155	70 (45.2)	46 (35-56)	31 (44.3)	23 (10-47)	85 (54.8)	61 (51-70)	55 (64.7)	46 (22-106)
Z. C, Feng et al[4]	Jan.17-Feb.1, 2020	China	141	126 (89.4)	41 (33-52)	65 (51.6)	19.2 (8.9-24.8)	15 (10.6)	58 (44-66)	7 (46.7)	44 (25-51.6)
L, Mao et al[7]	Jan.16-19, 2020	China	214	126 (58.9)	48.9±14.7	43 （34.1）	9.4 (0.4-126)	88 (41.1)	58.2±15	44 （50）	37.1 (0.1-212)
J. Y, Liu et al[10]	Jan.13-31, 2020	China	61	44 (72.1)	41 (1-76)	21 (47.7)	10.3 (3.6-21.9)	17 (27.9)	56 (34-73)	10 (58.8)	23.5 (2.7-71.2)
J, Liu et al[11]	Jan.5-24, 2020	China	40	27 (67.5)	43.2±12.3	8 (29.6)	7.6 (3.1-57.3)	13 (32.5)	59.7±10.1	7 (53.8)	62.9 (42.4-86.6)
Z, Wang et al[12]	Jan.16-29, 2020	China	69	55 (79.7)	37 (32-51)	25 (45)	11.3 (6.5-26.3)	14 (20.3)	70 (62-77)	7 (50)	81.5 (49-106)
K, Li et al[13]	Jan.-Feb. 2020	China	83	58 (69.9)	41.9 (10.6)	29 (50)	9.6 (2.1-29.9)	25 (30.1)	53.7 (12.3)	15 (60)	89.2 (48-135)
S, Wan et al[14]	Jan.23-Feb.8, 2020	China	135	95 (70.4)	44 (33-49)	52 (54.7)	7.7 (1.9-31.1)	40 (29.6)	56 (52-73)	21 (52.5)	91 (52.7-136.3)
S. J, Yan et al[15]	Jan.22-Mar.13, 2020	China	168	132 (78.6)	49 (34-60)	60 (45.5)	11.8 (1.2-26.2)	36 (21.4)	61 (50-68)	21 (58.3)	33.8 (18.5-64.4)

[a] Data are presented as median (IQR) or mean (SD); [b] Dare are presented as median (IQR); CRP, C-reactive protein; NA, not available;

Table S3. Procalcitonin.

Studies	Date	Country	Sample size	Non-severe patients				Severe patients			
				n (%)	Age(yrs) [a]	Men (%)	PCT (ug/L) [b]	n (%)	Age (yrs) [a]	Men (%)	PCT (ug/L) [b]
J. J, Zhang et al[1]	Jan.16-Feb.3, 2020	China	140	82 (58.6)	51.5 (26-78)	38 (46.3)	0.05 (0.03-0.1)	58 (41.4)	64 (25-87)	33 (56.9)	0.1 (0.06-0.3)
Y. L, Liu et al[5]	Jan.2-Feb.1, 2020	China	109	56 (51.4)	49 (37-59)	31 (55.4)	0.06 (0.05-0.09)	53 (48.6)	61 (52-70)	28 (52.8)	0.15 (0.08-0.37)
H. Z, Lu et al[6]	Jan.1- Feb.9, 2020	China	265	243 (91.7)	NA	NA	0.03 (0.02-0.05)	22 (8.3)	NA	NA	0.08 (0.05-0.15)
S. J, Yan et al[15]	Jan.22-Mar.13, 2020	China	168	132 (78.6)	49 (34-60)	60 (45.5)	0.04 (0.02-0.06)	36 (21.4)	61 (50-68)	21 (58.3)	0.07 (0.03-0.26)
Z, Wang et al[12]	Jan.16-29, 2020	China	69	55 (79.7)	37 (32-51)	25 (45)	0.13 (0.13-0.15)	14 (20.3)	70 (62-77)	7 (50)	0.13 (0.13-0.15)
K, Li et al[13]	Jan.-Feb. 2020	China	83	58 (69.9)	41.9 (10.6)	29 (50)	0.04 (0.03-0.07)	25 (30.1)	53.7 (12.3)	15 (60)	0.09 (0.05-0.22)
S, Wan et al[14]	Jan.23-Feb.8, 2020	China	135	95 (70.4)	44 (33-49)	52 (54.7)	0.04 (0.03-0.06)	40 (29.6)	56 (52-73)	21 (52.5)	0.11 (0.08-0.16)
Lei, L et al[16]	Jan.20-Feb.3, 2020	China	51	44 (86.3)	44 (33-49)	28 (63.7)	0.04 (0.03-0.06)	7 (13.7)	52 (44-60)	4 (57.1)	0.1 (0.05-0.24)

[a] Data are presented as median (IQR) or mean (SD); [b] Dare are presented as median (IQR); PCT, Procalcitonin; NA, not available;

Table S4. D-dimer.

Studies	Date	Country	Sample size	Non-severe patients				Severe patients			
				n (%)	Age(yrs) [a]	Men (%)	D-dimer (μg/mL) [b]	n (%)	Age (yrs) [a]	Men (%)	D-dimer (μg/mL) [b]
J. J, Zhang et al[1]	Jan.16-Feb.3, 2020	China	140	82 (58.6)	51.5 (26-78)	38 (46.3)	0.2 (0.1-0.3)	58 (41.4)	64 (25-87)	33 (56.9)	0.4 (0.2-2.4)
H. Z, Lu et al[6]	Jan.1- Feb.9, 2020	China	265	243 (91.7)	NA	NA	0.39 (0.28-0.72)	22 (8.3)	NA	NA	0.8 (0.5-3.5)
L, Mao et al[7]	Jan.16-19, 2020	China	214	126 (58.9)	48.9±14.7	43 (34.1)	0.4 (0.2-8.7)	88 (41.1)	58.2±15	44 (50)	0.9 (0.1-20)
S. J, Yan et al[15]	Jan.22-Mar.13, 2020	China	168	132 (78.6)	49 (34-60)	60 (45.5)	0.3 (0.2-0.5)	36 (21.4)	61 (50-68)	21 (58.3)	0.6 (0.3-2.6)
S, Wan et al[14]	Jan.23-Feb.8, 2020	China	135	95 (70.4)	44 (33-49)	52 (54.7)	0.3 (0.2-0.5)	40 (29.6)	56 (52-73)	21 (52.5)	0.6 (0.4-1.1)
Lei, L et al[16]	Jan.20-Feb.3, 2020	China	51	44 (86.3)	44 (33-49)	28 (63.7)	0.28 (0.18-0.46)	7 (13.7)	52 (44-60)	4 (57.1)	0.6 (0.28-1.4)
J, Liu et al[11]	Jan.5-24, 2020	China	40	27 (67.5)	43.2±12.3	8 (29.6)	0.4 (0.2-0.8)	13 (32.5)	59.7±10.1	7 (53.8)	0.9 (0.7-1.5)

[a] Data are presented as median (IQR) or mean (SD); [b] Dare are presented as median (IQR); NA, not available.

Table S5. The eigenvector centrality- and degree enrichment-value of genes.

Gene types	Gene Symbol	Eigenvector centrality	Degree enrichment
Stroke-associated genes	ACTA2	0.05	0.03
	ALOX5AP	0.03	0.90
	APOB	0.05	0.39
	CD59	0.04	0.53
	COL4A2	0.04	0.05
	FGA	0.03	0.17
	FGL1	0.05	0.39
	GH1	0.13	0.82
	KIF5B	0.09	0.47
	LTC4S	0.03	0.54
	NOTCH3	0.06	0.35
	NPPA	0.03	0.04
	SERPINA5	0.04	0.52
	SCNN1A	0.05	0.86
	VCAM1	0.63	0.62
	VWF	0.03	0.42
	MFN2	0.04	0.75
	BCAP31	0.04	0.18
	DDAH1	0.04	0.84
	SAMHD1	0.12	0.81
Overlap genes	GLA	0.13	0.73
	GOLGB1	0.15	0.70
	HMOX1	0.13	0.44
SARS-CoV-2 target genes	ACTB	0.16	0.33
	ANXA2	0.16	0.84
	ARF1	0.14	0.87
	ARF6	0.10	0.47
	BCL2L1	0.09	0.61
	CAV1	0.09	0.36
	CD9	0.09	1.03
	AP2M1	0.14	0.33
	CSNK2B	0.17	0.80
	DDX5	0.14	0.30
	DNMT1	0.14	0.62
	EEF1A1	0.16	0.41
	MARK2	0.10	0.61

	STOM	0.14	0.59
	GNB1	0.14	0.69
	GSK3A	0.10	1.04
	GSK3B	0.12	0.50
	HNRNPA1	0.14	-0.04
	HNRNPA2B1	0.14	0.37
	HSPD1	0.14	0.19
	ITGB1	0.14	0.56
	JUN	0.12	0.07
	KPNB1	0.16	0.60
	KPNA2	0.14	0.39
	LOX	0.10	1.11
	SMAD3	0.09	0.07
	MOV10	0.10	0.00
	NCL	0.14	0.25
	NONO	0.14	0.45
	FURIN	0.10	1.35
	PFDN5	0.10	0.43
	PHB	0.14	0.52
	PLAT	0.10	1.56
	POR	0.10	1.20
	PPIA	0.15	0.95
	PPP1CA	0.14	0.10
	PPT1	0.14	1.12
	PSMA2	0.10	0.58
	PSMC2	0.10	0.53
	PTBP1	0.14	0.59
	RAB1A	0.10	0.74
	RAB5C	0.14	0.56
	UPF1	0.14	0.44
	RPS20	0.14	0.47
	SKP2	0.10	0.45
	TLE3	0.09	0.83
	UBE2I	0.11	0.31
	VCP	0.15	0.39
	EIF4H	0.16	1.19
	XPO1	0.14	-0.39
	CSDE1	0.10	0.76
	REEP5	0.09	0.79
	BAG6	0.14	0.24

	CUL2	0.12	0.53
	EIF3I	0.16	0.93
	RIPK1	0.09	0.64
	PABPC4	0.14	0.67
	HGS	0.09	0.17
	DDX21	0.16	0.76
	CEP135	0.10	0.48
	UBAP2L	0.14	0.98
	RBX1	0.10	0.45
	G3BP1	0.14	0.56
	PPIH	0.14	1.16
	SYNCRIP	0.14	0.20
	HYOU1	0.09	0.91
	RAB10	0.14	1.05
	TWF2	0.09	0.89
	SPART	0.10	1.05
	MDN1	0.14	0.99
	PMPCA	0.09	0.91
	NUP210	0.14	1.16
	LARP1	0.14	0.61
	DDAH2	0.15	1.41
	PABPC1	0.14	0.36
	CHMP2A	0.14	1.08
	ERLEC1	0.10	1.21
	MAT2B	0.10	1.09
	GOLGA7	0.14	1.65
	TRMT1	0.10	0.98
	SELENOS	0.09	1.29
	ERGIC1	0.10	1.28
	GCC1	0.10	0.78
	TUBB6	0.14	0.79
	CHMP4B	0.14	0.70
	HNRNPA3	0.14	0.65

Table S6. The Log-fold change (logFC) and adjusted p-value (q) of all disease genes in nasal tissues (GEO ID: GSE152075) from COVID-19 positive patients and controls.

Type	Genes	logFC	q
Virus entry	ACE2	2.38492508	7.00E-08
Virus entry	TMPRSS2	-1.3944161	1.30E-06
Virus entry	NRP1	0.84729629	0.01941728
Virus entry	FURIN	-0.6631013	0.05162535
Stroke~COVID-19 Network	JUN	-3.2157248	1.52E-30
Stroke~COVID-19 Network	CD9	-2.7979571	4.50E-20
Stroke~COVID-19 Network	CD59	-2.2649247	9.41E-19
Stroke~COVID-19 Network	POR	-2.3248914	1.02E-16
Stroke~COVID-19 Network	RPS20	-1.2299662	1.83E-12
Stroke~COVID-19 Network	EEF1A1	-0.9241661	1.89E-11
Stroke~COVID-19 Network	EIF4H	-1.3547383	8.43E-10
Stroke~COVID-19 Network	PFDN5	-1.1598956	8.68E-09
Stroke~COVID-19 Network	SELENOS	-1.8498964	1.49E-08
Stroke~COVID-19 Network	SCNN1A	-0.9889621	1.52E-07
Stroke~COVID-19 Network	PPIA	-0.9054351	2.58E-07
Stroke~COVID-19 Network	PABPC4	-0.9435768	1.80E-06
Stroke~COVID-19 Network	CHMP4B	-1.0572118	2.28E-06
Stroke~COVID-19 Network	PPP1CA	-1.3422252	4.21E-06
Stroke~COVID-19 Network	UPF1	-1.1268732	5.32E-06
Stroke~COVID-19 Network	TRMT1	-1.6927994	7.10E-06
Stroke~COVID-19 Network	HNRNPA3	0.81414546	7.76E-06
Stroke~COVID-19 Network	AP2M1	-0.9539931	8.75E-06
Stroke~COVID-19 Network	NOTCH3	-1.4262853	1.70E-05
Stroke~COVID-19 Network	DDX5	-0.6485753	2.78E-05
Stroke~COVID-19 Network	ACTB	-0.6557513	3.70E-05
Stroke~COVID-19 Network	ITGB1	-0.839848	3.75E-05
Stroke~COVID-19 Network	DDAH2	-1.4357708	5.33E-05
Stroke~COVID-19 Network	ANXA2	-0.5300466	6.03E-05
Stroke~COVID-19 Network	BCL2L1	-0.8914472	0.00013904
Stroke~COVID-19 Network	ARF1	-0.7374288	0.00015946
Stroke~COVID-19 Network	KPNB1	0.66617251	0.00019467
Stroke~COVID-19 Network	PHB	-0.9018536	0.00031991
Stroke~COVID-19 Network	BAG6	-0.9015113	0.00046227
Stroke~COVID-19 Network	GH1	0.49223538	0.00056056

Stroke~COVID-19 Network	LTC4S	-0.6151903	0.00071209
Stroke~COVID-19 Network	GNB1	-0.5794367	0.00118441
Stroke~COVID-19 Network	FGA	1.06297776	0.00136935
Stroke~COVID-19 Network	EIF3I	-0.735242	0.00163463
Stroke~COVID-19 Network	PABPC1	-0.4553943	0.00197259
Stroke~COVID-19 Network	FGL1	1.01466442	0.00220779
Stroke~COVID-19 Network	UBE2I	-0.8318314	0.00230239
Stroke~COVID-19 Network	HGS	-0.7534479	0.00232613
Stroke~COVID-19 Network	PSMC2	0.7193255	0.00293506
Stroke~COVID-19 Network	PTBP1	-0.6538127	0.00567565
Stroke~COVID-19 Network	HYOU1	-0.7499448	0.00717318
Stroke~COVID-19 Network	APOB	1.566146	0.00718935
Stroke~COVID-19 Network	ALOX5AP	1.0728783	0.01083944
Stroke~COVID-19 Network	HNRNPA2B1	0.3715083	0.0153573
Stroke~COVID-19 Network	RBX1	-0.7591541	0.01745408
Stroke~COVID-19 Network	TUBB6	-0.8857258	0.02501092
Stroke~COVID-19 Network	MOV10	0.63529241	0.026397
Stroke~COVID-19 Network	NONO	-0.4312954	0.02767419
Stroke~COVID-19 Network	GSK3A	-0.6196112	0.03306906
Stroke~COVID-19 Network	TWF2	-0.70429	0.03610062
Stroke~COVID-19 Network	REEP5	-0.4454237	0.03668402
Stroke~COVID-19 Network	VWF	-0.8534377	0.03907774
Stroke~COVID-19 Network	PMPCA	-0.6915166	0.03924082
Stroke~COVID-19 Network	DDX21	0.47396463	0.04126143
Stroke~COVID-19 Network	ERLEC1	-0.547723	0.04194391
Stroke~COVID-19 Network	CSNK2B	0.44928395	0.05038953
Stroke~COVID-19 Network	MFN2	-0.4855852	0.06100622
Stroke~COVID-19 Network	VCP	-0.3962876	0.06179744
Stroke~COVID-19 Network	GOLGB1	-0.3427962	0.07073286
Stroke~COVID-19 Network	VCAM1	0.96629971	0.08733804
Stroke~COVID-19 Network	XPO1	0.29890508	0.0887235
Stroke~COVID-19 Network	RAB1A	-0.4006356	0.08990183
Stroke~COVID-19 Network	DDAH1	-0.4242289	0.09517691
Stroke~COVID-19 Network	RAB10	0.35147541	0.10974471
Stroke~COVID-19 Network	RIPK1	0.36875769	0.11097745
Stroke~COVID-19 Network	SPART	0.41093713	0.14693429
Stroke~COVID-19 Network	CSDE1	0.22804218	0.15013478
Stroke~COVID-19 Network	CHMP2A	-0.3525881	0.158582
Stroke~COVID-19 Network	UBAP2L	0.25005257	0.18839167
Stroke~COVID-19 Network	ERGIC1	-0.3077426	0.20258027

Stroke~COVID-19 Network	RAB5C	-0.3076464	0.25110299
Stroke~COVID-19 Network	GLA	0.4622751	0.28042401
Stroke~COVID-19 Network	MDN1	0.27563463	0.28086988
Stroke~COVID-19 Network	LOX	-0.3811247	0.29100216
Stroke~COVID-19 Network	PSMA2	-0.292646	0.29267603
Stroke~COVID-19 Network	PPT1	0.36852124	0.30125362
Stroke~COVID-19 Network	HSPD1	-0.2397411	0.3064349
Stroke~COVID-19 Network	PLAT	-0.42201	0.31170371
Stroke~COVID-19 Network	SERPINA5	0.51569658	0.34762842
Stroke~COVID-19 Network	ACTA2	0.36578757	0.3890912
Stroke~COVID-19 Network	DNMT1	-0.2297787	0.39727739
Stroke~COVID-19 Network	CAV1	-0.4641878	0.40244461
Stroke~COVID-19 Network	NUP210	-0.3398387	0.42315136
Stroke~COVID-19 Network	LARP1	0.15008993	0.45488345
Stroke~COVID-19 Network	GSK3B	0.1765598	0.45592709
Stroke~COVID-19 Network	PPIH	0.30643804	0.51268135
Stroke~COVID-19 Network	G3BP1	0.1368071	0.51639996
Stroke~COVID-19 Network	KPNA2	0.19856887	0.54504125
Stroke~COVID-19 Network	ARF6	0.13778928	0.55606103
Stroke~COVID-19 Network	SAMHD1	0.37690455	0.5572859
Stroke~COVID-19 Network	NCL	0.12962277	0.57040895
Stroke~COVID-19 Network	BCAP31	0.30057684	0.5714995
Stroke~COVID-19 Network	KIF5B	-0.0991677	0.61462343
Stroke~COVID-19 Network	HMOX1	0.16863591	0.67526452
Stroke~COVID-19 Network	GCC1	-0.1500696	0.71494623
Stroke~COVID-19 Network	SMAD3	0.07016559	0.79235014
Stroke~COVID-19 Network	SYNCRIP	-0.0629757	0.79458655
Stroke~COVID-19 Network	SKP2	-0.0903962	0.83806769
Stroke~COVID-19 Network	GOLGA7	0.06839941	0.84596832
Stroke~COVID-19 Network	CEP135	-0.076118	0.84950104
Stroke~COVID-19 Network	MARK2	-0.0526102	0.86591357
Stroke~COVID-19 Network	CUL2	-0.029861	0.93531913
Stroke~COVID-19 Network	MAT2B	0.01985997	0.94828447
Stroke~COVID-19 Network	HNRNPA1	-0.0166929	0.94842603
Stroke~COVID-19 Network	STOM	0.01501625	0.96206906
Stroke~COVID-19 Network	TLE3	0.02024444	0.96485382
Stroke~COVID-19 Network	COL4A2	0.02199308	0.98410721
Stroke~COVID-19 Network	NPPA	0.65688044	0.99653413

Table S7. The Log-fold change (logFC) and adjusted p-value (q) of all disease genes in the published peripheral blood mononuclear cell samples (GEO ID: GSE157103) from COVID-19 positive patients vs. controls.

Type	Gene symbols	logFC	q
Virus entry	FURIN	-0.3800632	0.0211285
Virus entry	NRP1	-0.2450825	0.51037133
Stroke~COVID-19 Network	TWF2	-1.0659831	2.02E-09
Stroke~COVID-19 Network	KPNA2	0.54364382	3.16E-09
Stroke~COVID-19 Network	HMOX1	-1.0234854	8.02E-09
Stroke~COVID-19 Network	ACTB	-0.8092909	3.28E-08
Stroke~COVID-19 Network	CAV1	2.41129878	6.80E-07
Stroke~COVID-19 Network	BCAP31	-0.675672	2.17E-06
Stroke~COVID-19 Network	POR	-1.0082189	2.63E-06
Stroke~COVID-19 Network	PFDN5	-0.7993165	3.74E-06
Stroke~COVID-19 Network	CHMP2A	-0.7196042	6.89E-06
Stroke~COVID-19 Network	CHMP4B	-0.4958897	8.00E-06
Stroke~COVID-19 Network	LARP1	0.6060984	8.64E-06
Stroke~COVID-19 Network	CSNK2B	-0.5086987	9.70E-06
Stroke~COVID-19 Network	ITGB1	0.63711053	2.07E-05
Stroke~COVID-19 Network	PPP1CA	-0.5969161	2.27E-05
Stroke~COVID-19 Network	RPS20	-0.9151139	2.92E-05
Stroke~COVID-19 Network	BAG6	-0.312689	6.06E-05
Stroke~COVID-19 Network	HGS	-0.4473493	0.00012094
Stroke~COVID-19 Network	GOLGB1	0.42160045	0.00015639
Stroke~COVID-19 Network	ERLEC1	0.46905788	0.00017656
Stroke~COVID-19 Network	REEP5	-0.2945397	0.00020519
Stroke~COVID-19 Network	TRMT1	-0.7580788	0.00091773
Stroke~COVID-19 Network	RAB5C	-0.3075799	0.00099593
Stroke~COVID-19 Network	HSPD1	0.31741463	0.0013665
Stroke~COVID-19 Network	LTC4S	-1.3297942	0.0016006
Stroke~COVID-19 Network	AP2M1	-0.3129663	0.00178527
Stroke~COVID-19 Network	DDAH2	-0.7673638	0.00194124
Stroke~COVID-19 Network	JUN	0.73735184	0.00226797
Stroke~COVID-19 Network	PABPC1	-0.2938676	0.00277717
Stroke~COVID-19 Network	MDN1	0.64678859	0.00285277
Stroke~COVID-19 Network	EEF1A1	-0.375265	0.00295258
Stroke~COVID-19 Network	UBAP2L	0.32312942	0.00331425
Stroke~COVID-19 Network	XPO1	0.41525363	0.0061631

Stroke~COVID-19 Network	CUL2	0.22625853	0.00836698
Stroke~COVID-19 Network	KIF5B	0.42817263	0.0087663
Stroke~COVID-19 Network	TLE3	-0.4027262	0.00908945
Stroke~COVID-19 Network	DDX21	0.49499731	0.0116476
Stroke~COVID-19 Network	PSMC2	0.25221599	0.01217619
Stroke~COVID-19 Network	GCC1	0.29416465	0.0129936
Stroke~COVID-19 Network	MAT2B	0.30970376	0.01368362
Stroke~COVID-19 Network	GSK3A	-0.1945724	0.01699134
Stroke~COVID-19 Network	EIF3I	-0.2740488	0.02084741
Stroke~COVID-19 Network	ARF1	-0.2064657	0.02529819
Stroke~COVID-19 Network	PPT1	-0.3720942	0.02664833
Stroke~COVID-19 Network	SYNCRIP	0.26611855	0.0272145
Stroke~COVID-19 Network	PMPCA	-0.29514	0.02759448
Stroke~COVID-19 Network	HYOU1	0.32805309	0.03331428
Stroke~COVID-19 Network	PHB	-0.3479524	0.03617861
Stroke~COVID-19 Network	G3BP1	0.21563078	0.04091928
Stroke~COVID-19 Network	MARK2	-0.2288516	0.04370968
Stroke~COVID-19 Network	MOV10	0.68655513	0.04647197
Stroke~COVID-19 Network	DNMT1	0.35578412	0.04834338
Stroke~COVID-19 Network	TUBB6	-0.4992752	0.04952575
Stroke~COVID-19 Network	ANXA2	-0.2545929	0.05045944
Stroke~COVID-19 Network	CEP135	0.3857829	0.07573579
Stroke~COVID-19 Network	PPIH	-0.2705399	0.08096223
Stroke~COVID-19 Network	MFN2	0.34279561	0.09324806
Stroke~COVID-19 Network	EIF4H	-0.1169311	0.10128216
Stroke~COVID-19 Network	ARF6	0.08650291	0.11680528
Stroke~COVID-19 Network	RAB1A	0.23701876	0.12685945
Stroke~COVID-19 Network	KPNB1	0.28347804	0.12932035
Stroke~COVID-19 Network	SKP2	0.12791552	0.13412755
Stroke~COVID-19 Network	SMAD3	0.28545085	0.1352078
Stroke~COVID-19 Network	UPF1	-0.2172068	0.14486742
Stroke~COVID-19 Network	PTBP1	-0.130794	0.17931669
Stroke~COVID-19 Network	HNRNPA3	0.09800853	0.21095337
Stroke~COVID-19 Network	NOTCH3	-0.4385314	0.22560799
Stroke~COVID-19 Network	DDX5	0.1507848	0.23547053
Stroke~COVID-19 Network	STOM	0.33256187	0.27963828
Stroke~COVID-19 Network	RIPK1	0.1055012	0.28926001
Stroke~COVID-19 Network	PABPC4	-0.147194	0.29631462
Stroke~COVID-19 Network	VCP	-0.0710406	0.34748562
Stroke~COVID-19 Network	SPART	0.15620567	0.36939846

Stroke~COVID-19 Network	HNRNPA2B1	0.07341202	0.4001979
Stroke~COVID-19 Network	GOLGA7	0.12544034	0.40638911
Stroke~COVID-19 Network	NCL	0.09653495	0.40870702
Stroke~COVID-19 Network	ALOX5AP	-0.2088105	0.41185141
Stroke~COVID-19 Network	NONO	-0.0420438	0.45747921
Stroke~COVID-19 Network	SELENOS	0.10209979	0.46246481
Stroke~COVID-19 Network	CSDE1	0.07240452	0.4640905
Stroke~COVID-19 Network	PSMA2	0.07038767	0.48356196
Stroke~COVID-19 Network	GSK3B	0.11433748	0.4988884
Stroke~COVID-19 Network	GNB1	-0.0553848	0.50528941
Stroke~COVID-19 Network	HNRNPA1	-0.0831663	0.51294271
Stroke~COVID-19 Network	UBE2I	0.04119115	0.54625814
Stroke~COVID-19 Network	NUP210	0.11939546	0.55019446
Stroke~COVID-19 Network	SCNN1A	-0.1314882	0.58111067
Stroke~COVID-19 Network	RAB10	0.0869615	0.59250981
Stroke~COVID-19 Network	PPIA	-0.0715349	0.60950918
Stroke~COVID-19 Network	SAMHD1	0.09663703	0.64172045
Stroke~COVID-19 Network	VWF	-0.1258008	0.64707116
Stroke~COVID-19 Network	CD59	-0.1138806	0.70583774
Stroke~COVID-19 Network	ERGIC1	0.08146167	0.74194428
Stroke~COVID-19 Network	RBX1	-0.0425073	0.8318832
Stroke~COVID-19 Network	ACTA2	0.02779678	0.91739533
Stroke~COVID-19 Network	CD9	0.01889921	0.94571124
Stroke~COVID-19 Network	BCL2L1	-0.0115017	0.96133202
Stroke~COVID-19 Network	GLA	-0.0040958	0.97572493

Table S8. The Log-fold change (logFC) and adjusted p-value (q) of all disease genes in iPSC - cardiomyocytes (GEO ID: GSE150392) after SARS-CoV-2 infection.

Type	Gene Symbols	logFC	q
Virus entry	ACE2	-3.9070395	0.01234915
Virus entry	FURIN	1.22696493	0.01877172
Virus entry	NRP1	-0.4758719	0.3379689
Stroke~COVID-19 Network	TLE3	1.79803378	0.00282265
Stroke~COVID-19 Network	LOX	2.87277776	0.00316703
Stroke~COVID-19 Network	PLAT	1.81352148	0.00337
Stroke~COVID-19 Network	STOM	1.5955169	0.00378574
Stroke~COVID-19 Network	SAMHD1	3.08162018	0.00447378
Stroke~COVID-19 Network	PTBP1	1.43525443	0.00461668
Stroke~COVID-19 Network	JUN	2.38128457	0.00533026
Stroke~COVID-19 Network	NPPA	-3.2142683	0.00748652
Stroke~COVID-19 Network	ERGIC1	1.21478346	0.00774057
Stroke~COVID-19 Network	GSK3B	1.23140403	0.01125486
Stroke~COVID-19 Network	CD9	1.57882029	0.01728874
Stroke~COVID-19 Network	HNRNPA1	-0.9654769	0.01969186
Stroke~COVID-19 Network	SMAD3	1.34669239	0.02012609
Stroke~COVID-19 Network	ALOX5AP	1.68066976	0.02556975
Stroke~COVID-19 Network	SPART	1.2559052	0.02561814
Stroke~COVID-19 Network	TRMT1	0.85677973	0.02588827
Stroke~COVID-19 Network	GLA	1.02191668	0.02821373
Stroke~COVID-19 Network	DDAH1	0.85573106	0.02964649
Stroke~COVID-19 Network	VCAM1	1.3880442	0.02981453
Stroke~COVID-19 Network	ARF6	0.82341902	0.03343247
Stroke~COVID-19 Network	RIPK1	1.78486428	0.03385895
Stroke~COVID-19 Network	NOTCH3	1.5334222	0.03415622
Stroke~COVID-19 Network	MOV10	1.06888999	0.03850029
Stroke~COVID-19 Network	GNB1	0.96293938	0.03893087
Stroke~COVID-19 Network	RBX1	-1.6570299	0.04294341
Stroke~COVID-19 Network	CAV1	-1.3059941	0.04604122
Stroke~COVID-19 Network	HMOX1	1.24866871	0.04621388
Stroke~COVID-19 Network	LARP1	0.7255305	0.04708215
Stroke~COVID-19 Network	REEP5	-0.8294393	0.04778882
Stroke~COVID-19 Network	DDX21	0.64540906	0.05402248
Stroke~COVID-19 Network	CHMP4B	-1.285158	0.05557213
Stroke~COVID-19 Network	PPIH	-1.2382785	0.06063297
Stroke~COVID-19 Network	RPS20	-1.3426451	0.0679747

Stroke~COVID-19 Network	ACTA2	-1.0425816	0.06911177
Stroke~COVID-19 Network	EEF1A1	-0.8143044	0.07273564
Stroke~COVID-19 Network	XPO1	1.14014803	0.074608
Stroke~COVID-19 Network	PHB	-0.9093102	0.07664884
Stroke~COVID-19 Network	DDAH2	-0.7789382	0.10778822
Stroke~COVID-19 Network	TWF2	0.68246741	0.11212059
Stroke~COVID-19 Network	CHMP2A	-0.8673735	0.11374041
Stroke~COVID-19 Network	PFDN5	-1.0301947	0.11630691
Stroke~COVID-19 Network	HGS	0.50895421	0.11932136
Stroke~COVID-19 Network	ANXA2	0.66853862	0.12280911
Stroke~COVID-19 Network	NUP210	-1.0834537	0.12665586
Stroke~COVID-19 Network	RAB10	0.55295463	0.12902963
Stroke~COVID-19 Network	UPF1	1.16732958	0.13782495
Stroke~COVID-19 Network	KPNA2	-0.4614898	0.15408881
Stroke~COVID-19 Network	ITGB1	0.78053759	0.16125299
Stroke~COVID-19 Network	COL4A2	1.00706145	0.18024209
Stroke~COVID-19 Network	ACTB	0.59032445	0.1905811
Stroke~COVID-19 Network	MFN2	-0.8357756	0.19274281
Stroke~COVID-19 Network	GOLGB1	0.60603958	0.19341498
Stroke~COVID-19 Network	UBE2I	-0.5935882	0.20026909
Stroke~COVID-19 Network	GCC1	1.15869036	0.20132316
Stroke~COVID-19 Network	POR	0.37158685	0.20256291
Stroke~COVID-19 Network	UBAP2L	0.59947835	0.21960787
Stroke~COVID-19 Network	ERLEC1	0.3931005	0.21979764
Stroke~COVID-19 Network	BCL2L1	0.40226081	0.22515525
Stroke~COVID-19 Network	MARK2	0.40152036	0.22576253
Stroke~COVID-19 Network	HNRNPA3	-0.3472914	0.28086032
Stroke~COVID-19 Network	CD59	0.38787236	0.30005697
Stroke~COVID-19 Network	HYOU1	0.69930197	0.30342116
Stroke~COVID-19 Network	PSMC2	-0.3263875	0.30751181
Stroke~COVID-19 Network	HNRNPA2B1	-0.3461025	0.30941755
Stroke~COVID-19 Network	AP2M1	-0.3446236	0.31355397
Stroke~COVID-19 Network	HSPD1	-0.3122357	0.31406809
Stroke~COVID-19 Network	NCL	0.41168958	0.32935621
Stroke~COVID-19 Network	SYNCRIP	0.26689484	0.35460052
Stroke~COVID-19 Network	PPIA	-0.5617097	0.35980326
Stroke~COVID-19 Network	CSNK2B	-0.5235854	0.36872289
Stroke~COVID-19 Network	CEP135	0.30351613	0.37042791
Stroke~COVID-19 Network	G3BP1	0.33437003	0.374438
Stroke~COVID-19 Network	PPP1CA	-0.3592528	0.39096225

Stroke~COVID-19 Network	KPNB1	0.28070476	0.41100071
Stroke~COVID-19 Network	PABPC4	-0.3020708	0.43827893
Stroke~COVID-19 Network	SELENOS	0.32056402	0.50222956
Stroke~COVID-19 Network	GOLGA7	-0.2475369	0.50811252
Stroke~COVID-19 Network	VCP	0.34170553	0.54885117
Stroke~COVID-19 Network	RAB1A	-0.2120182	0.58584119
Stroke~COVID-19 Network	TUBB6	-0.2099747	0.60739943
Stroke~COVID-19 Network	SKP2	0.1924566	0.61940591
Stroke~COVID-19 Network	PMPCA	-0.1282792	0.67535284
Stroke~COVID-19 Network	GSK3A	-0.2138336	0.67863246
Stroke~COVID-19 Network	PABPC1	0.16229089	0.68294135
Stroke~COVID-19 Network	DDX5	0.16215929	0.70227133
Stroke~COVID-19 Network	MDN1	0.14392406	0.72367333
Stroke~COVID-19 Network	ARF1	0.140349	0.76017417
Stroke~COVID-19 Network	MAT2B	-0.1047056	0.77539671
Stroke~COVID-19 Network	CSDE1	-0.1511455	0.79677839
Stroke~COVID-19 Network	CUL2	0.09544237	0.80437729
Stroke~COVID-19 Network	PSMA2	-0.152759	0.80541204
Stroke~COVID-19 Network	KIF5B	0.08205581	0.82671538
Stroke~COVID-19 Network	EIF4H	0.06777419	0.83850746
Stroke~COVID-19 Network	PPT1	0.05188411	0.86503742
Stroke~COVID-19 Network	DNMT1	-0.0406858	0.91128806
Stroke~COVID-19 Network	RAB5C	-0.0573654	0.91484562
Stroke~COVID-19 Network	BCAP31	-0.0401269	0.93260032
Stroke~COVID-19 Network	NONO	0.03115795	0.93887539
Stroke~COVID-19 Network	EIF3I	-0.0330988	0.94022887
Stroke~COVID-19 Network	BAG6	-0.018353	0.9715565

References

1. Zhang JJ, Dong X, Cao YY, Yuan YD, Yang YB, Yan YQ, et al. Clinical characteristics of 140 patients infected with sars-cov-2 in wuhan, china. *Allergy*. 2020, 75(7):1730-1741.
2. Mo P, Xing Y, Xiao Y, Deng L, Zhao Q, Wang H, et al. Clinical characteristics of refractory covid-19 pneumonia in wuhan, china. *Clinical Infectious Diseases.* 2020, doi: 10.1093/cid/ciaa270
3. Wang D, Hu B, Hu C, Zhu F, Liu X, Zhang J, et al. Clinical characteristics of 138 hospitalized patients with 2019 novel coronavirus-infected pneumonia in wuhan, china. *JAMA*. 2020, 323(11):1061-1069.
4. Feng Z, Yu Q, Yao S, Luo L, Duan J, Yan Z, et al. Early prediction of disease progression in 2019 novel coronavirus pneumonia patients outside wuhan with ct and clinical characteristics. 2020:2020.2002.2019.20025296, doi: https://doi.org/10.1101/2020.02.19.20025296
5. Liu Y, Sun W, Li J, Chen L, Wang Y, Zhang L, et al. Clinical features and progression of acute respiratory distress syndrome in coronavirus disease 2019. 2020:2020.2002.2017.20024166, doi: https://doi.org/10.1101/2020.02.17.20024166
6. Lu H, Ai J, Shen Y, Li Y, Li T, Zhou X, et al. A descriptive study of the impact of diseases control and prevention on the epidemics dynamics and clinical features of sars-cov-2 outbreak in shanghai, lessons learned for metropolis epidemics prevention. 2020:2020.2002.2019.20025031, doi: https://doi.org/10.1101/2020.02.19.20025031
7. Mao L, Jin H, Wang M, Hu Y, Chen S, He Q, et al. Neurologic manifestations of hospitalized patients with coronavirus disease 2019 in wuhan, china. *JAMA Neurol*. 2020, 77(6):683-690.
8. Wang Y, Zhou Y, Yang Z, Xia D, Geng S. Clinical characteristics of patients with

severe pneumonia caused by the 2019 novel coronavirus in wuhan, china. Respiration. 2020, doi: 10.1159/000507940

9. Guan WJ, Ni ZY, Hu Y, Liang WH, Ou CQ, He JX, et al. Clinical characteristics of coronavirus disease 2019 in china. *The New England Journal of Medicine*. 2020, doi: 10.1056/NEJMoa2002032

10. Liu J, Liu Y, Xiang P, Pu L, Xiong H, Li C, et al. Neutrophil-to-lymphocyte ratio predicts critical illness patients with 2019 coronavirus disease in the early stage. *Journal of Translational Medicine*. 2020;18:206

11. Liu J, Li S, Liu J, Liang B, Wang X, Wang H, et al. Longitudinal characteristics of lymphocyte responses and cytokine profiles in the peripheral blood of sars-cov-2 infected patients. *EBioMedicine*. 2020;55:102763

12. Wang Z, Yang B, Li Q, Wen L, Zhang R. Clinical features of 69 cases with coronavirus disease 2019 in wuhan, china. *Clinical Infectious Diseases*. 2020, 71(15):769-777.

13. Li K, Wu J, Wu F, Guo D, Chen L, Fang Z, et al. The clinical and chest ct features associated with severe and critical covid-19 pneumonia. *Investigative radiology*. 2020;55:327-331

14. Wan S, Xiang Y, Fang W, Zheng Y, Li B, Hu Y, et al. Clinical features and treatment of covid-19 patients in northeast chongqing. *Journal Medical Virology*. 2020, 92(7):797-806.

15. Yan S, Song X, Lin F, Zhu H, Wang X, Li M, et al. Clinical characteristics of coronavirus disease 2019 in hainan, china. 2020:2020.2003.2019.20038539

16. lei l, Jian-ya G. Clinical characteristics of 51 patients discharged from hospital with covid-19 in Chongqing，China. 2020:2020.2002.2020.20025536
